# Pollution impacts on water bugs (Nepomorpha, Gerromorpha): state of the art and their biomonitoring potential

**DOI:** 10.1007/s10661-022-09961-2

**Published:** 2022-03-28

**Authors:** Gábor Bakonyi, Tamás Vásárhelyi, Borbála Szabó

**Affiliations:** 1 Department of Zoology and Ecology, Hungarian University of Agriculture and Life Sciences, 2100 Gödöllő, Hungary; 2 Budapest, Hungary; 3 Centre for Ecological Research, Institute of Ecology and Botany, “Lendület” Landscape and Conservation Ecology, 2163 Vácrátót, Hungary

**Keywords:** Ecotoxicology, Pesticide, Heavy metal, Light pollution, Salinisation, Eutrophication, Species sensitivity distribution

## Abstract

As water pollution poses an increasing risk worldwide, it is timely to assess the achievements of the aquatic macroinvertebrate ecotoxicology to provide a sound basis for the discipline’s future and support the development of biomonitoring. Aquatic and semi-aquatic bugs (Hemiptera: Nepomorpha, Gerromorpha) are ubiquitous in almost all water types, sometimes in high densities, and play a significant role in organic material turnover and energy flow. Nevertheless, they are ignored in the water pollution biomonitoring schemes. Here, based on 300 papers, we review and evaluate the effects of chemical pesticides, microorganism-derived pesticides, insecticides of plant origin, heavy metals, eutrophication, salinisation and light pollution which are summarised for the first time. Our review encompasses the results of 100 laboratory and 39 semi-field/field experiments with 47 pesticides and 70 active ingredients. Pyrethroids were found to be more toxic than organochlorine, organophosphate and neonicotinoid insecticides to water bugs, like other macroinvertebrate groups. Additionally, in 10 out of 17 cases, the recommended field concentration of the pesticide was higher than the LC50 values, indicating potential hazards to water bugs. The recommended field concentrations of pesticides used in mosquito larvae control were found non-toxic to water bugs. As very few replicated studies are available, other findings on the effects of pesticides cannot be generalised. The microorganism-derived pesticide Bti appears to be safe when used at the recommended field concentration. Data indicates that plant-derived pesticides are safe with a high degree of certainty. We have identified three research areas where water bugs could be better involved in water biomonitoring. First, some *Halobates* spp. are excellent, and *Gerris* spp. are promising sentinels for Cd contamination. Second, Micronecta and, to a certain extent, Corixidae species composition is connected to and the indicator of eutrophication. Third, the species composition of the Corixidae is related to salinisation, and a preliminary method to quantify the relationship is already available. Our review highlights the potential of water bugs in water pollution monitoring.

**Supplementary Information:**

The online version contains supplementary material available at 10.1007/s10661-022-09961-2.

## Introduction

Aquatic (Nepomorpha) and semi-aquatic (Gerromorpha) bugs are ubiquitous in freshwater biotopes, with some marine species too. They play an essential role in the functioning of aquatic ecosystems. Predators have strong top-down effects on food chains (Rumschlag et al., 2020) and connect aquatic and terrestrial ecosystems (Schriever & Lytle, 2020). Some predatory taxa as *Anisops* spp., Belostomatidae, *Gerris* spp., Nepidae and Notonectidae species are effective mosquito control agents in various regions of the world (Dambach, 2020; Medlock & Snow, 2008; Shaalan & Canyon, 2009). The density of water bugs can sometimes be very high. For example, summer corixids density in Hungarian soda pans was higher than 2000 individuals/m^2^ (Bakonyi, 1983; Cozma et al., 2020). The economic importance of the water bugs is also remarkable because, besides effective mosquito control, they are a food source for several vertebrates (including humans), used for fish and poultry feeding, and pests in breeding ponds of fish fry (Papácek, 2013).

Pollutants substantially affect the aquatic and semi-aquatic macroinvertebrate community, but currently, little is known about the causal links between pollution and the ecological condition of a water habitat (Backhaus et al., 2019). Concerning food webs that include water bugs, it was found that besides direct toxicity on a bug population, toxic agents induced a shift in aquatic food web structure (Schrama et al., 2017).

The morphologically as well as biologically highly diverse aquatic and semi-aquatic Heteroptera’s global species number is similar to that of Ephemeroptera, Odonata and Plecoptera (Balian et al., 2007), commonly used taxa in water pollution assessments. However, water bugs are far less involved in these schemes than the mentioned other three macroinvertebrate groups. For example, Vitecek et al. (2021) list 11 taxon richness and 23 indicator group metrics used for Ecological Status Class Assessment Tools for European rivers and lakes. Out of the 34 metrics, only one is related to Heteroptera, while 14 to Ephemeroptera, 4 to Odonata and 11 to Plecoptera, respectively. Brasil et al. (2020), based on an extensive search for environmental predictors, found that Heteroptera was involved in 1.9% of the papers, while the EPT group (Ephemeroptera, Plecoptera, Trichoptera) was in 21.6%. Moreover, Carter et al. (2017) discussed aquatic macroinvertebrates as bioindicators but did not mention water bugs at all. All these data are consistent with the general opinion that the proportions of taxa included in ecotoxicological studies do not represent the proportions of taxa found in nature (Prosser et al., 2021).

Although little attention has been paid to water bugs as bioindicators, some new data suggest that it is time to review and reassess the role of water bugs in water pollution biomonitoring. Just to mention two of them, a recent study based on species sensitivity distribution analysis shows that Corixidae and Notonectidae are among the most sensitive macroinvertebrates to imidacloprid and diazinon (Becker et al., 2020). Furthermore, Gerromorpha species are suitable for monitoring new types of pollutants, such as diluted bitumen and surface washing agents (Black et al., 2021).

It is legitimate to ask why are water bugs (Nepomorpha and Gerromorpha) a neglected group among the macroinvertebrates in water assessment and biomonitoring schemes? Ecotoxicological studies on water bugs have not yet been reviewed, and information on this topic is very scattered. Therefore, further questions may arise: (1) are there enough data available for the use of water bugs in biomonitoring? (2) Are the data consistent enough to draw general conclusions about their usefulness in water pollution biomonitoring?

To clarify these questions, we aim to review the results of a vast amount of pollution studies with water bugs (Nepomorpha and Gerromorpha) and see if they were used for biomonitoring purposes. More specifically, we examine and evaluate how pesticides, heavy metals, eutrophication, salinisation and light pollution affect water bug populations and communities. These are those research areas where most papers are available about water bug ecotoxicology. Besides the direct effects, we will pay special attention to how interactions between species influence the effects of pollutants. Finally, after a comprehensive assessment of the data, we recommend issues where water bugs could be more involved in water pollution biomonitoring and suggest promising research areas.

## Materials and methods

### Literature search

Papers were searched with the following search codes among peer-reviewed journal papers in the Web of Science (WoS, core collection): ALL = (“water bug” OR backswimmer OR Notonect* OR Corixid* OR Ranatra OR Micronecta OR “Plea minutissima” OR Sigara OR Anisops OR Lethocerus OR Belostom* OR “water strider” OR Gerri* OR Hydrometra) AND ALL = ( ecotoxicology OR pollution OR pesticide OR bactericide OR fungicide OR herbicide OR insecticide OR antibiotics OR carbamate OR organophosphorus OR organochlorine OR neonicotinoid OR pyrethroid OR “pesticide mixture” OR spinosyn OR “bacillus thuringiensis” OR “heavy metal” OR “plant extract” OR “mosquito control” OR eutrophication OR salinisation OR “light pollution”) AND ALL = (“acute toxicity” OR “sublethal-effects” OR “dose–response” OR LC50 OR EC50 OR “species richness” OR diversity OR abundance OR density OR community OR “species sensitivity distribution” OR behaviour) and Scopus TITLE-ABS-KEY(“water bug” OR backswimmer OR Notonect* OR Corixid* OR Ranatra OR Micronecta OR “Plea minutissima” OR Sigara OR Anisops OR Lethocerus OR Belostom* OR “water strider” OR Gerri* OR Hydrometra) AND ALL (ecotoxicology OR pollution OR pesticide OR bactericide OR fungicide OR herbicide OR insecticide OR antibiotics OR carbamate OR organophosphorus OR organochlorine OR neonicotinoid OR pyrethroid OR “pesticide mixture” OR spinosyn OR “bacillus thuringiensis” OR “heavy metal” OR “plant extract” OR “mosquito control” OR eutrophication OR salinisation OR “light pollution”) AND TITLE-ABS-KEY(“acute toxicity” OR “sublethal-effects” OR “dose–response” OR LC50 OR EC50 OR “species richness” OR diversity OR abundance OR density OR community OR “species sensitivity distribution” OR behaviour). The search was finished on 30.05.2021. The reference list of the collected papers was checked for additional relevant publications not found during the WoS and Scopus search.

### Processing of the papers

Titles and abstracts of the collected papers were checked whether they contained relevant data about water bugs for this paper’s purposes. We discarded the paper if it did not contain relevant information based on the title and abstract.

Papers were evaluated whether (1) it focused on aquatic-semi-aquatic Heteroptera ecotoxicology, or at least these taxa were involved decisively in the study in the case of multispecies studies, (2) methods were described in a repeatable way and (3) appropriate statistical analyses were performed on the data. Only those studies were regarded that reported sample size, mean and variance for all treatment groups in question as a measurement of quality of the study.

We excluded articles from the final collection for various reasons. In some earlier studies, other than current metrics were used (e.g. LC_100_ instead of LC_50_). Papers were omitted if an apparent technical error was found (e.g. confused data units, no control mortality given). The taxon name was misused in some other cases, showing that the author is not profoundly familiar with water bug taxonomy. Occasionally, methods were described in an unreproducible way, or the statistical analysis description was lacking or specified incorrectly. Only papers in English were considered. Even if they had an English summary, papers written in other languages were excluded from this review because we could not check the methods in detail. Only original peer-reviewed papers were included in our study to avoid accidental mistakes from inadequate citing by review papers, books or reports (Gurevitch et al., 2001). Finally, we excluded papers that used experimental results from other papers for further calculations (e.g. papers presenting SSD curves calculated on data of other studies).

We found 481 papers with relevant ecotoxicological data on aquatic and semi-aquatic bugs; 181 were excluded because of one or more of the criteria mentioned above. The remaining 300 papers were included in this overview. Data collected from these papers are presented in the supplementary material (Tables S1–S6).

Original data for a dose–response study were presented, but LC_50_ values were not calculated in a paper, we determined it (“Quest Graph™ LC50 Calculator” *AAT Bioquest, Inc*, 19 Apr. 2021, https://www.aatbio.com/tools/lc50-calculator). Data from graphs were obtained using the WebPlotDigitizer-4.4 software (https://automeris.io/WebPlotDigitizer).

The sensitivity of water bugs to different pesticide groups (Fig. 3) was tested with the non-parametric Kruskal–Wallis test, and if a significant main effect was found, Dunn’s post hoc test was performed with the Past 4.03 software (Hammer et al., 2001). A more detailed statistical test on a specific insecticide or active ingredient effect on a particular species was not possible due to the small sample size.

Species sensitivity distribution (SSD) analyses were performed to find species-specific sensitivity differences for pesticide classes, with the software by Thorley and Schwarz (2018). Only LC_50_ data (Table S1) were considered for these analyses because of the small number of appropriate EC_50_ values. If multiple LC_50_ values were available for a given species, their geometric mean was applied.

### Pesticide effects on the water bugs

#### Chemical pesticides

A total of 34 papers on laboratory dose–response studies were found. In these studies, 26 water bug species, 21 pesticides and 37 active substances were tested in 100 experiments. Unfortunately, no replicated study was performed under identical circumstances (same laboratory). Table 1 shows that some pesticides are toxic to water bugs at concentrations recommended for field use. In 10 cases out of the 17 studies, the LC_50_ values were lower than the recommended concentration by the manufacturer. This finding indicates that the use of many pesticides can be dangerous for water bugs.

**Table 1:** Comparison of LC_50_ values to the recommended field concentration to see whether the suggested application concentration may have a potential danger to the studied water bug population. LC_50_ (µg/L): average and the minimum–maximum or 95% confidence limit. Concentration: recommended field concentration based on information from the manufacturer or distributor. *: LC_50_ is lower than the recommended field concentration

Species	Pesticide	LC_50_ (µg/L)	Concentration (µg/L)	Author
Corixa punctata	Dursban 4E	2.0 (1.5–2.6)	0.03–1.2	van Wijngaarden et al. (1993)
Anisops sardeus	Sumithion	8.61 (7.8–9.3)	0.025–0.13	Lahr et al. (2001)
Anisops sardeus	Dursban	0.90 (0.88–0.92)	0.03–1.2	Lahr et al. (2001)
Anisops sardeus	Fyfanon	42.2 (40.5–44.9)	0.68–1.1	Lahr et al. (2001)
Anisops sardeus	Ficam*	373 (275–567)	5000	Lahr et al. (2001)
Anisops sardeus	Decis*	0.012 (0.010–0.014)	0.3–1.5	Lahr et al. (2001)
Anisops sardeus	Karate*	0.025 (0.023–0.031)	0.4–0.5	Lahr et al. (2001)
Anisops sardeus	Bulldock*	0.019 (0.015–0.025)	20	Lahr et al. (2001)
Anisops sardeus	Dimilin	1937 (1800–2020)	0.1–0.4	Lahr et al. (2001)
Anisops sardeus	Nomolt	249 (233–267)	0.75	Lahr et al. (2001)
Anisops sardeus	Alsystin*	189 (168–228)	200–400	Lahr et al. (2001)
Notonecta glauca	Karate*	0.016 (0.008–0.036)	0.4–0.5	Schroer et al. (2004)
Buenoa tarsalis	Decis 25EC*	0.004 (0.003 –0.006)	0.25–1.2	Gutiérrez et al. (2017a)
Martarega bentoi	Decis 25EC*	0.103 (0.039 –0.228)	0.25–1.2	Gutiérrez et al. (2017a)
Diplonychus rusticus	Temper EC*	50 (1–90)	250–2000	Reegan et al. (2020)
Diplonychus rusticus	Superkiller 10%EC	100 (10–290)	0.54–0.74	Reegan et al. (2020)
Diplonychus rusticus	Baton 5*	20 (1–07)	833–1500	Reegan et al. (2020)

**Fig. 1:**
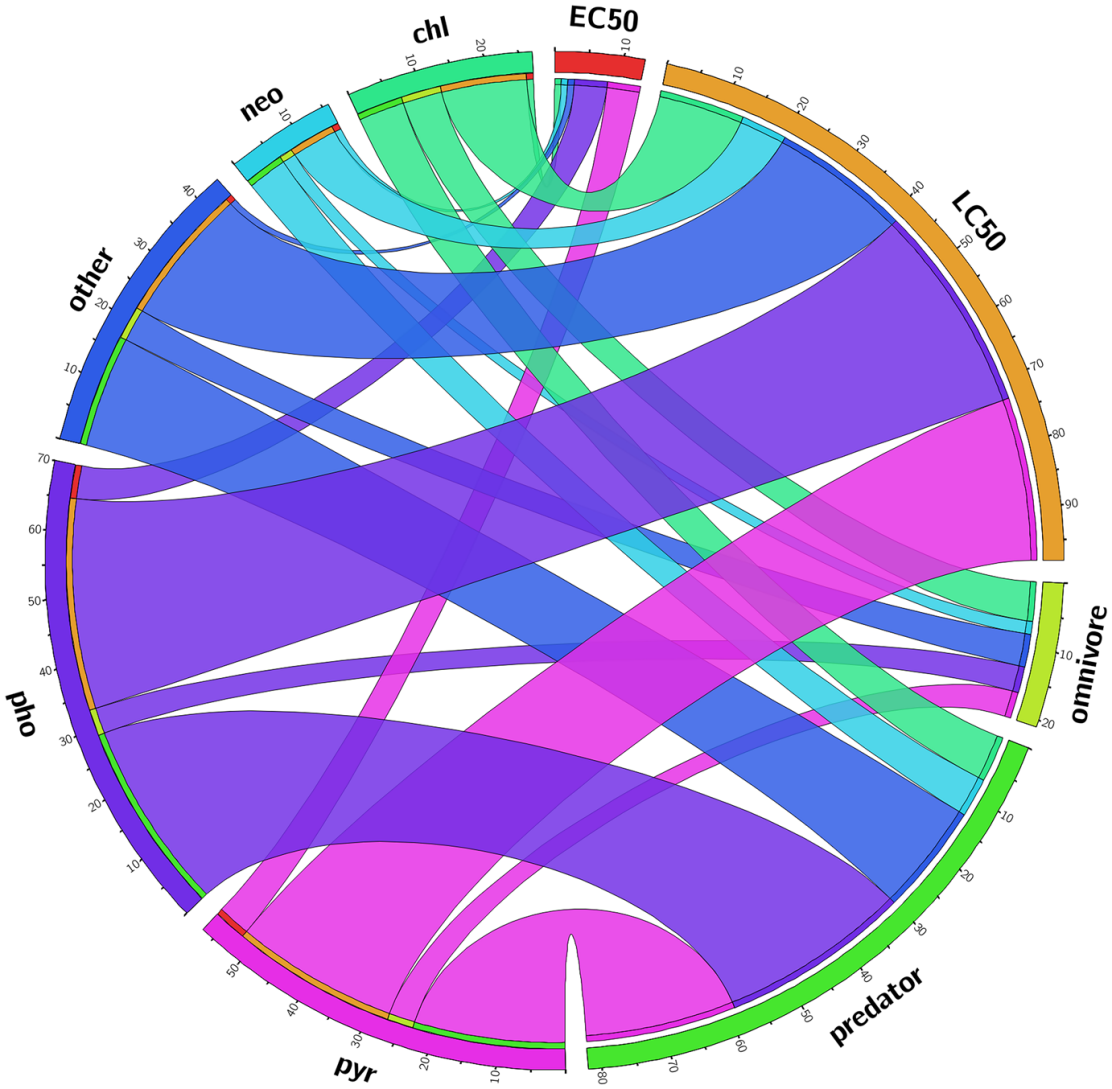
The relationship between pesticides, feeding type of the tested water bugs, and test types based on results of the laboratory studies. A thicker line indicates that more tests have been performed with the components that are connected than where there is only a thin line between the components. chl: organochloride, neo: neonicotinoid, other: all other pesticide types not included into named ones, pho: organophosphate, pyr: pyrethroid

**Fig. 2:**
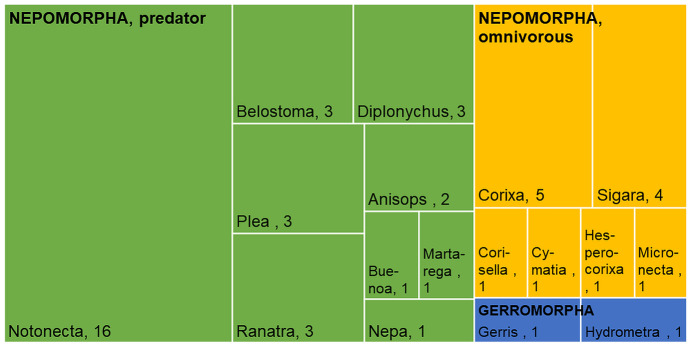
This tree map shows how often species of a given genus are tested with pesticides in laboratory studies. If more than one test has been carried out on a species in a given paper, it is only included once. The colours indicate taxonomic and feeding categories (Gerromorpha are predators). Genus names are given. The size of each rectangle is based on the number of experiments (if a toxicological study was conducted with one pesticide but with several species, the number of data is considered as the number of species tested). Conversely, if one species was tested but with more than one pesticide, the number of pesticides was considered)

Most laboratory LC_50_ studies were performed with organophosphate and pyrethroid insecticides (Fig. 1). A reasonably large group of “other” insecticides shows the great diversity of the tested materials (Table S1). In addition, considerably more predators (or a predator with more pesticides) than omnivores were tested. Predators were tested mainly in order to calculate LC_50_ values (Fig. 1). Moreover, the number of EC_50_ values is much lower than that of the LC_50_ values (Fig. 1). The results are comparable only in exceptional cases because of the differences in test duration, end-point, laboratory and the diversity of the species and insecticides used in the studies (Table S1). As Fig. 2 shows, almost exclusively Nepomorpha species were studied in the experiments, and predators were tested more often than omnivores. Notonecta species were most commonly used in the tests, followed by Corixa and Sigara species.

**Fig. 3:**
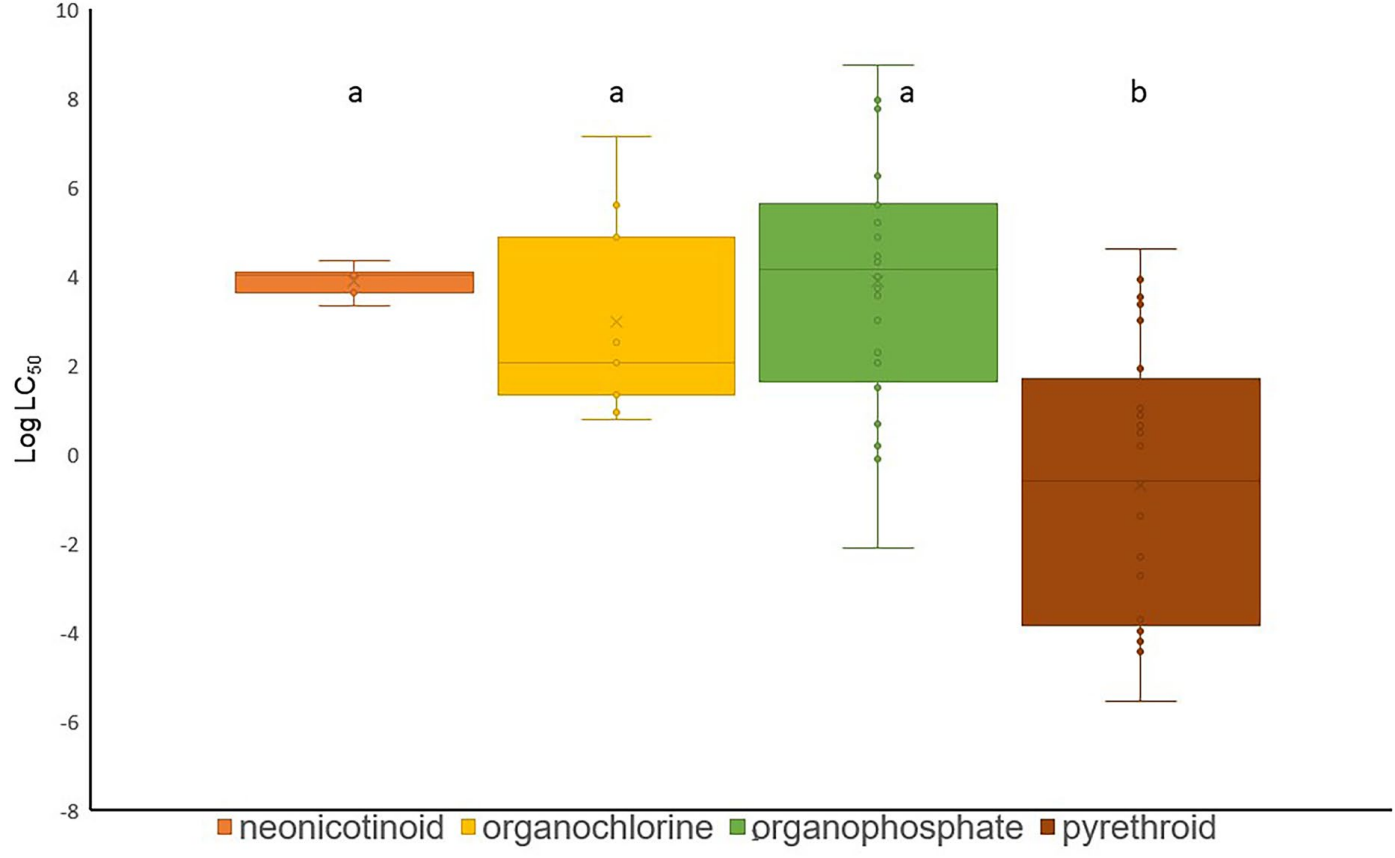
The sensitivity of water bugs to different pesticide groups. Means marked with different letters are significantly different from each other (*p* < 0.05). The boxes show the minimum, maximum, sample median and the first and third quartiles

Sufficient data are available to compare the sensitivity of water bugs to four major insecticide groups. Like other macroinvertebrate groups (Halstead et al., 2015), water bugs have been proven significantly more sensitive to pyrethroids than organochlorine, neonicotinoid and organophosphate insecticides (Kruskal–Wallis *H*: 24.2, *p* < 0.001). The effects of neonicotinoids, organochlorines and organophosphates differ from pyrethroids by a significance level of 0.007, 0.011 and < 0.001, respectively (Fig. 3).

Based on the data in Table S1, species-specific statements can be made in some cases. For example, although carbamates LC_50_ values are similar to that of organophosphate insecticides if all data are compared, carbamates are less toxic to *Notonecta undulata* (Federle & Collins, 1976) and *Anisops sardeus* (Lahr et al., 2001) than organophosphates. Moreover, Mian and Mulla (1992) found that the order of toxicity for aquatic invertebrates is permethrin < cypermethrin < deltamethrin. Deltamethrin is approximately two orders of magnitude more toxic than permethrin (Table S1). However, the cypermethrin data are highly variable.

Insecticides usually occur as mixtures in the environment. Although mixture effects would be essential for environmental risk assessments (de Souza Machado et al., 2019), only a few mixture toxicity data are available. For example, synergism was found if Volaton (i.e. propoxur, carbamate) and Unden (i.e. phoxim, organophosphate) insecticide effects were tested on *A. sardeus* (Lahr et al., 2001). The effect of the commercial formulation of Excel Endohyper (endosulfan 35% + cypermethrin 5%) on *Diplonychus rusticus* was negligible, despite the presence of the pyrethroid (Kalimuthu et al., 2010). The study of chemical mixture effects is neglected in water bug ecotoxicology, similarly to other macroinvertebrates.

Several papers deal with the insecticide effects on the vector mosquito larvae at the recommended field concentration under semi-field or field conditions. In a few cases, simultaneously, their side effect is tested on non-target taxa, including the water bugs. Miura and Takahashi (1974) applied Altosid (i.e. S-methoprene), an insect growth regulator and TH 6040, a juvenile hormone analogue, as mosquito control agents. Altosid did not influence the *Notonecta unifasciata* and *Buenoa* spp. population density, but after TH 6040 application, no reproduction of the bugs was observed. Furthermore, lambda-cyhalothrin applied against mosquito larvae decreased the Notonectidae and Corixidae density (Lawler et al., 2007). Bioactive compounds of *Solanum nigrum* (Solanaceae) did not enhance the *Diplonychus annulatum* (Belostomatidae) mortality (Rawani et al., 2017). In addition, silver nanoparticle toxicity (synthesised with the plant *Nicandra physalodes*) was almost two orders of magnitude more toxic for three mosquito species than for the non-target *Diplonychus indicus* water bug (Govindarajan et al., 2016). These data show that except for the TH 6040 (i.e. of the insecticides Dimilin, Labyrinth, Termigrad, etc.), all tested insecticides are safe for water bugs in the recommended field concentration. However, we should note that the recommended field concentration data vary within a wide range in some cases.

The community-level responses of macroinvertebrates to chemical pesticide impacts in surface waters provide ecologically relevant data for environmental risk assessment (Liess & Von Der Ohe, 2005) and biomonitoring (Menezes et al., 2010). There are many relevant microcosmos and field studies available. We collected data from the literature on pesticide effects on water bugs found at the community level (Table S2a). One concentration (typically the recommended field concentration) was applied in these studies.

**Fig. 4:**
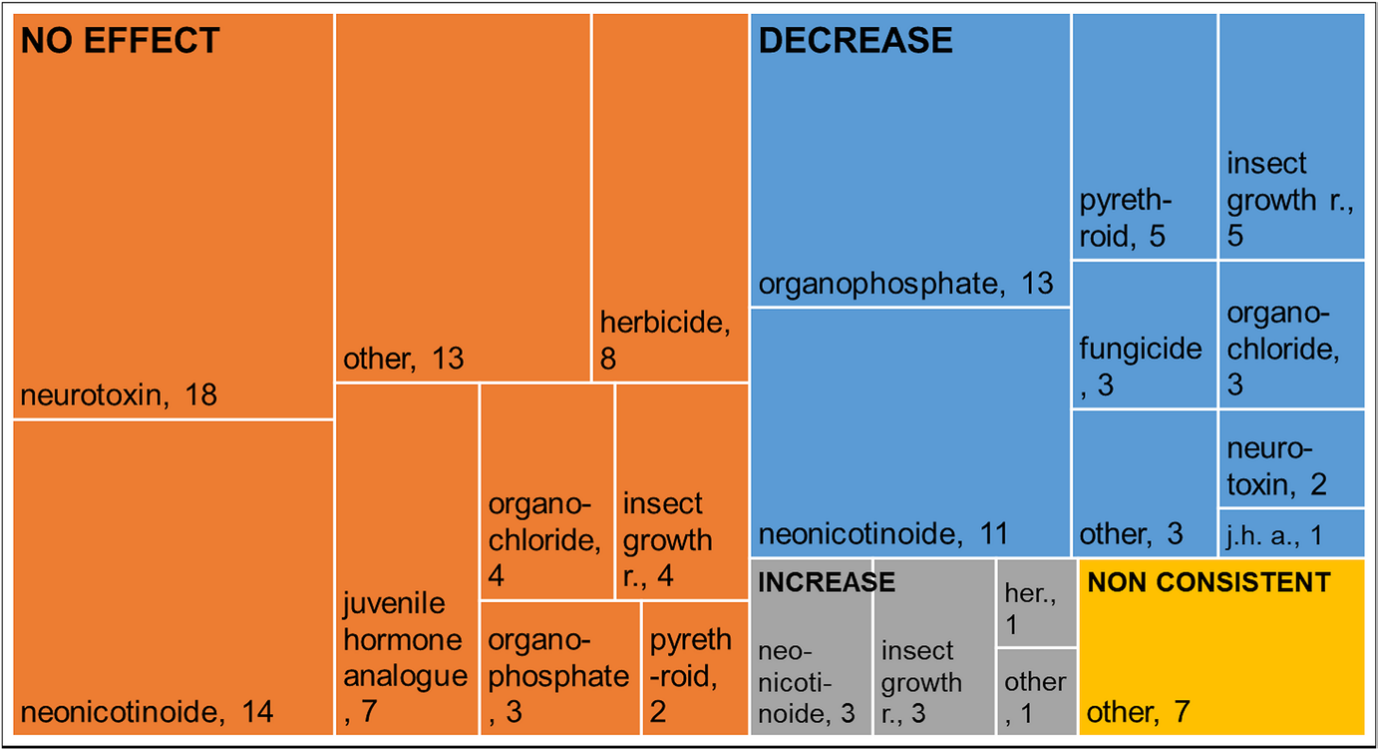
The tree map shows the pesticide effects on the density of water bugs based on the community level analyses. The colours indicate pesticide groups. The size of each rectangle is based on the number of experiments. The number of data represents the number of communities tested. The effects of pesticides on communities have mostly been studied using recommended field concentrations. In most cases no effect was found on the density of water bug species; in several cases, density decreased, and in a few cases, density increased due to the pesticide application. Numbers: papers in which the effect of a given pesticide occurred. insect growth r.: insect growth regulator, j.h.a.: juvenile hormone analogue, her.: herbicide

In most cases (58%), the treatments did not affect the water bugs (Fig. 4). When a paper described an effect, the treatment usually reduced the density of the species studied (36%). In a few cases, pesticide treatment increased the densities (6%). No clear trends can be identified for individual pesticide groups. Neonicotinoids, for example, reduce or increase water bug densities but often have no effect. Similar LC_50_ values were obtained in the laboratory and the field in a few cases (Table S2b). The sample number is only three; therefore, no generalisation can be made.

We have to point out that the macroinvertebrate community studies are frequently not suitable for detecting the insecticide effects on water bugs because direct effects on a given group (like water bugs) are not separable from the overall and indirect effects. Moreover, the taxonomic resolution is usually coarse (e.g. family or genus level identification is typical) and/or the water bug density is low. Consequently, these studies are not suitable for a detailed analysis of the reactions of the water bug assemblages.

Water bug assemblages per se are rarely used for water pollution assessment. Zinc and manganese concertations were negatively related to water bug diversity and richness (Ishadi et al., 2014). It was found that intensively managed (including use of pesticides) oil palm farming alongside Amazonian streams decreased water bug richness (Cunha et al., 2015). In addition, *Rhagovelia brunae* density increased while the richness decreased, probably due to decreasing competition pressure (Cunha & Juen, 2017). In Brazil, Giehl et al. (2018) found six species associated with the most preserved sites, which did not occur in the disturbed habitats. All of these species belonged to the Naucoridae family.

Agricultural land cover (which may be a marker of pesticides used in crop protection) was negatively correlated with densities of *Corisella tarsalis*, *C. inscripta*, *Trichocorixa calva* and *Sigara decorata* in stormwater ponds (Foltz & Dodson, 2009). On the other hand, a study in the Netherlands did not find a correlation between *Micronecta scholtzi*, *Plea minutissima* and *Sigara striata* density and imidacloprid concentration of the surface waters (Van Dijk et al., 2013). Moreover, pesticide effect on density may be masked by immigration of the species with a good flying ability (Whatley et al., 2015). However, no such effect was found in a water bug–dominated macroinvertebrate community by Trekels et al. (2011a) in a distance-isolated mesocosm experiment. Therefore, the data presented show that no general conclusions can be drawn, except for the decline of species number due to pesticide load. The issue is worth further research.

#### Microorganism-derived pesticides

Insecticides manufactured from *Bacillus thuringiensis israeliensis* (Bti) and *B. sphaericus* effectively decrease mosquito larvae population density and are used worldwide for mosquito control (Lacey, 2007). That is why side effect studies were performed, and data for water bugs are available. Laboratory experiments were focused on specified water bug species, which proved to be effective predators of mosquito larvae under natural circumstances. Bti insecticides applied at a field concentration recommended against mosquito larvae did not enhance the water bug mortality (Table S3). Aly and Mulla (1987) fed *Notonecta undulata* with Bt-intoxicated mosquito larvae. They did not find Bti effect on several life-history parameters, but the predation rate decreased significantly. In another study, no such effect was detected on *Notonecta* sp. and *Diplonychus indicus* (Gunasekaran et al., 2004). Contrary to the previous result, *Buenoa tarsalis* mortality was not influenced by Bt-Horus SC insecticide, but Bt-exposed individuals consumed more *Aedes aegypti* larvae than the unexposed ones after 24-h recovery (Gutiérrez et al., 2017a).

The side effects of Bti insecticide application on macroinvertebrates (including water bugs) were investigated to detect changes in population density and traits (Table S3). Bti insecticides (different formulations) did not influence the field density of predatory aquatic and semi-aquatic bug species (Garcia et al., 1981; Marina et al., 2014; Sebastien & Brust, 1981) or the omnivore *Corisella* spp. (Miura et al., 1980). One study showed a significant reduction of the *Notonecta indica* population due to Bti (Purcell, 1981). However, this repeatedly cited study is based on a limited number of notonectids (three individuals before and zero after the Bti application). Consequently, it seems to be an unreliable finding. Although some issues still need addressing, e.g. persistence, resistance and environmental risk assessment (Bruehl et al., 2020), Bti pesticides used in mosquito control seem safe for the water bug populations if the recommended field concentration is applied.

The insecticide spinosad is manufactured from the bacterium *Saccharopolyspora sinosa*. It effectively reduced the density of vector mosquito larvae (Santos & Pereira, 2020). However, a modest effect of spinosad at a field concentration was found on Notonectidae (Hertlein et al., 2010) and Corixidae (Lawler & Dritz, 2013) in mesocosm experiments.

Avermectins are derived from the soil actinomyces *Streptomyces avermitilis*. The water boatman *Hesperocorixa sahlbergi* and creeping water bug *Ilyocoris cimicoides* were resistant compared to other macroinvertebrates if avermectin-impregnated plant powder was applied against mosquito larvae. However, *I. cimicoides* mortality was significantly enhanced due to the insecticide application in a laboratory experiment (Belevich et al., 2021). In conclusion, Bt-insecticides seem safe based on the available data, but spinosad and avermectins have side effects on water bugs if the recommended field concentrations are applied.

#### Insecticides of plant origin

Natural extracts of plant origin are environmentally friendly options to traditional pest (including mosquito) control with synthetic compounds. However, these products’ ecotoxicological mechanisms need to be clarified regarding non-target organisms (Amichot et al., 2018). There is a comprehensive data set about plant-derived extracts on predatory water bugs (Table S4). It is straightforward that the toxicity effect is appreciably different depending on which extract was applied, alone or in a mixture. Consequently, there is a potential to choose less toxic pesticides for water bugs.

Several plant-derived extracts’ LC_50_ is much higher for water bugs than mosquito larvae, which these pesticides are intended to control (Baranitharan et al., 2020). It is essential to emphasise that the dataset is biased because almost all experiments were performed on two predatory species, *Anisops bouvieri* and *Diplonychus indicus*, and no other water bugs were tested (Table S4). Comparing the LC_50_ values of the chemical pesticides (Table S1) with plant extracts (Table S4), the toxic effect difference can be even as high as six orders of magnitude in some cases. Consequently, plant-originated pesticides are generally less toxic to non-target water bugs than chemical ones.

### Heavy metals and water bugs

Among the heavy metals, cadmium and mercury received special attention in water bug ecotoxicology. Both cadmium and mercury are inhibitors of some enzymes in higher concentrations. Laboratory dose–response studies showed considerable differences between Cd sensitivity of different species. For example, LC_50_ values of Cd for *Ranatra elongata* (Shukla et al., 1983) and *Anisops sardeus* (Chanu et al., 2017) were lower than that for *Corixa punctata* (Slooff, 1983) (Table S5a). Although few data are available, it seems that predators proved more sensitive to Cd in laboratory tests than the omnivorous *C. punctata*.

Like many other organisms, some water bug species can also accumulate heavy metals from the environment. Cheng et al. (1976) were the first who proved high Cd concentration up to 208 µg/g in the open ocean sea skater *Halobates sobrinus*. Bull et al. (1977) and Schulz-Baldes and Cheng (1980) found high Cd concentration in some *Halobates micans* populations as well (Table S5a). Compared to the highest Cd values in zooplankton, a factor of 10 or more has been found in *Halobates* spp. Surface film on the seawater is the primary source of Cd in *Halobates robustus* body, and it was hypothesised that population Cd concentration is related to oceanic surface water currents (Cheng, 1985; Schulz-Baldes & Cheng, 1979). The biomagnification of Cd in the *Halobates sericeus*–*Procelsterna cerulea* food chain has also been proven (Cheng et al., 1984).

Similarly to Halobates, high Cd concentration was found in four field-sampled freshwater Gerris species (*G. argentatus*, *G. lateralis*, *G. odontogaster*, *G. thoracicus*) in Finland (Lodenius et al., 1998). The Cd concentration of *Sigara* sp. in Eastern England rivers was low (Barak & Mason, 1989). The physiological reason for the high Cd concentration in the body of *Halobates* spp. and *Gerris* spp. is unknown (Nummelin et al., 2007; Schulz-Baldes & Cheng, 1981).

An interesting finding is that in laboratory studies, predators are more sensitive to cadmium than herbivores, whereas higher concentrations were measured in predators in field-collected specimens (Table S5a). We expected species that could accumulate higher concentrations of this heavy metal in their bodies to be more tolerant to Cd pollution (see, e.g. Tan & Wang, 2011). There can be some reasons why we failed to get this result: (1) the studies examined different species, (2) only those specimens can be collected on the field which can tolerate high concentrations or (3) because of other yet unknown reasons.

**Fig. 5:**
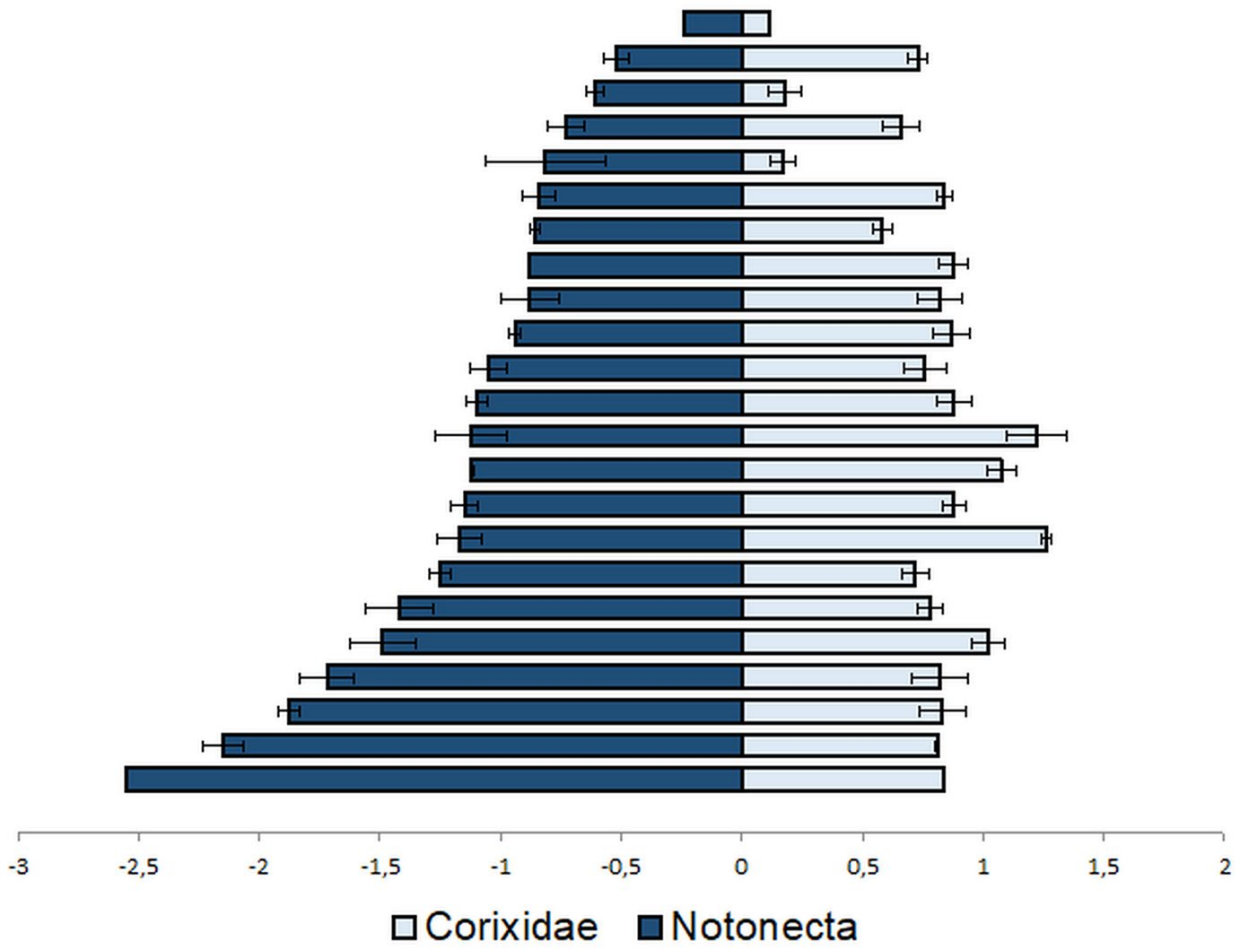
THg (µg/g dry weight; ± SD) concentrations of Notonecta and Corixidae species at 23 sampling sites. There are only three sites with higher THg concentrations in Corixidae species. Data from Cremona et al. (2008), Blackwell and Drenner (2009), Ackerman et al. (2010), Williams et al. (2011), Henderson et al. (2012). THg: total Hg

Mercury can be biomagnified mainly in its methylated form along the aquatic food chain. Consequently, mercury threatens water bug consumers, fishes, birds and even humans. Generally, the Hg concentration of the field sampled Heteroptera is often higher than the concentration of other macroinvertebrates (Allen et al., 2005; Cleckner et al., 1998; Cremona et al., 2008), but Chumchal et al. (2011) did not find a difference, comparing *Belostoma* sp. to dragonfly larvae. Based on an extensive dataset (Table S5b), another distinct pattern emerges: the Hg concentration seems to depend on the feeding type. For example, predatory Notonecta species usually have higher Hg concentrations than omnivorous Corixidae (Fig. 5). Another aspect is that Hg was notably more toxic to the omnivorous *Corixa punctata* than Cd (Slooff, 1983).

A significant correlation was found between the sediment and Hg concentration of *Micronecta scholtzi* (Agra et al., 2010) and *Sigara semistriata* and *S. fossarum* (Lindholm et al., 2014), showing that sediment is the primary Hg source, probably through food for these corixids. Correlation of the Hg concentration of Corixidae and black-winged stilt (Himantopus himantopus) chick feathers was found as a sign of a predator–prey relationship (Tavares et al., 2008). The importance of food in MeHg accumulation in aquatic heteropterans is emphasised by Tremblay et al. (1996).

Jardine et al. (2005) suggest Gerris species as mercury sentinels in small watercourses. Later on, however, it was found that the Hg in Gerris bodies has terrestrial origin to a significant extent (Jardine et al., 2009). Therefore, further studies are necessary to clarify whether Gerris are suitable sentinels or not.

Apart from cadmium and mercury, there are little data on other heavy metal and metalloid pollution effects on water bugs. Corixidae Se concentration is low compared to other insects (Saiki & Lowe, 1987; Schuler et al., 1990). This finding is explainable by the results of Thomas et al. (1999), who found that primary Se exposure of *Trichocorixa reticulata* is via food. Some corixids are feeding on low-Se sediment and detritus, which explain their low Se level. The Cu, Pb and Zn content of Sigara, Ranatra, Belostoma and Notonecta species is not appreciably different from other macroinvertebrates (Anderson, 1977; Anderson et al., 1978). Furthermore, Goodyear and McNeill (1999) suggest that macroinvertebrates generally do not biomagnify Cu and Zn in freshwater. Therefore, these elements are of little relevance to the ecotoxicology of water bugs.

### Eutrophication determines community composition

Intensive agriculture and growing urbanisation strongly influence aquatic ecosystems because nutrient inputs from run-off water increase eutrophication (Schindler, 2006). Increasing eutrophication causes a significant shift in the food composition of invertebrates and the structure of the food chain (van der Lee et al., 2021). Moreover, eutrophication changes the effects of pesticides and predators on target organisms (Oliveira dos Anjos et al., 2021). Consequently, trophic states will significantly change the density of water bugs or the species composition in a given water body.

First Macan (1954), then later several other authors provided data about species primarily sampled from eutrophic biotopes (Table S6a). According to Lock et al. (2013), water bug species composition is strongly influenced by the eutrophication level of waters in Belgium. The overview of the data shows that *Plea minutissima* and *Microvelia reticulata* seem to be indicators of eutrophic standing waters (Table S6a).

Several data supports the idea that the Micronecta species composition can be a suitable indicator of lake eutrophication. Jansson (1977a, b, 1987) performed detailed sampling series in Finland lakes of different trophic statuses. He suggested that some Micronecta species and their dominance relationship indicate the eutrophication status of lakes. Later on, some studies confirmed this statement (Kurzatkowska, 2003; Vásárhelyi & Bakonyi, 2012). Suitable indicators for oligotrophy is *Micronecta poweri* and for eutrophy *M. scholtzi* (Table S6b). So, the changes in the Micronecta fauna of a given area can be an early sign of the eutrophication of a water body. Therefore, the study of Micronecta species composition is recommended for biomonitoring purposes.

However, the problem is that Micronecta species (especially larvae and females) are difficult to identify. This problem will possibly be solved soon, as the barcoding of Nepomorpha and Gerromorpha is in an advanced state. Water bugs of Germany and adjacent regions were identified with high safety based on their DNA barcode sequences (82% of the analysed species) (Havemann et al., 2018). Weigand et al. (2019) revealed that about 92% of the European water bugs already have at least five DNA barcodes. Consequently, water bugs will probably be among the first taxa of all European macroinvertebrate species with full barcode coverage.

Although the species level is the desirable taxonomic resolution in several pollution assessment studies, it is not an indispensable condition for routine biomonitoring (Jones, 2008). For example, the Biological Monitoring Working Party Index score system is based on family-level identification of benthic macroinvertebrates. There are other new ways to avoid taxonomical (species-level identification) problems. The morpho-species concept or trait analysis is increasingly used for biomonitoring purposes (Rubach et al., 2012). Moreover, the robust identification methods based on machine learning are also increasingly promising (Blair et al., 2020). Considering these technical developments, a system for monitoring the lake eutrophication based on difficult-to-identify Micronecta assemblages of a given area can be proposed.

### Salinisation effects on non-marine water bugs

Salinisation is a major and increasing anthropogenic threat to freshwaters. It is predicted that many lakes in the USA will exceed the threshold limit in the next 50 years (Dugan et al., 2017). Although this is a severe anthropogenic effect, it received little attention in freshwater ecology (Cañedo-Argüelles et al., 2019; Dugan et al., 2017), but ecotoxicological studies of water bugs are extensive in this area. For many decades, field measurements of the water conductivity and salt concentration were frequently performed during water bug samplings. That is why there are respectable data available, primarily for the salt tolerance of corixids.

The global number of Corixidae species is almost seven hundred (Polhemus & Polhemus, 2008). Only a small proportion of them are living in saltwater. According to Scudder (1976), 57 species inhabit saline waters (salinity over 3 000 mg/L). It was found that Corixidae varies in their salinity optimum and tolerances, and the difference between species can be large. Some of them, such as *Sigara assimilis*, *S. selecta*, *Trichocorixa reticulata* and *T. verticalis*, are often collected in highly saline or hypersaline waters (Table S7). This finding may be related to the fact that *T. verticalis* can regulate the haemolymph ion composition in hypo- and hyperosmotic water (Jang & Tullis, 1980; Sanguinetti, 1980; Tones & Hammer, 1975), and the other species probably also have similar mechanisms. Osmoregulation studies have revealed the physiological backgrounds for *Corixa dentipes* (Staddon, 1964), *C. punctata* (Vangenechten et al., 1979), *Cenocorixa bifida* and *C. expleta* (Scudder et al., 1972), *C. blaisdelli* (Cooper et al., 1989) and *Corisella edulis* (Frick & Sauer, 1974), showing considerable osmoregulatory abilities of these species.

The tolerance limits of some Corixidae are already known. For example, *Sigara stagnalis* and *S. lateralis* (Broring & Niedringhaus, 1988; Van Vierssen & Verhoeven, 1983), (Lancaster & Scudder, 1987) and *T. verticalis* (Carbonell et al., 2020) are tolerant to water salinity, while the tolerance range of *Callicorixa praeusta* is narrow (Van Vierssen & Verhoeven, 1983). Because of the species differences in tolerance and salinity optimum, it is expected that changes in corixid assemblages would indicate changes in salinity. Savage (1994) has found that changes in the proportion of species in a corixid assemblage can be a good indicator of lake salinity changes and developed a model for monitoring. However, Savage’s method (Savage, 1994) has not come into general use.

A similar attempt to Savage’s method was also made with Micronecta species. Sites and Vitheepradit (2010) suggest Micronecta as indicators of salinity conditions. However, Zalizniak et al. (2006) did not find any effect of four different water salinity types on *Micronecta robusta* LC_50_ values. These results leave the question open whether changes in Micronecta assemblages are suitable for water salinity monitoring. Aquatic water bug species richness and salinity correlate negatively (Lancaster & Scudder, 1987), similarly to the species richness of the whole macroinvertebrate community in streams (Piscart et al., 2005; Velasco et al., 2006) and ponds (Sowa et al., 2020). Based on LC_50_ values, Ephemeroptera and aquatic Heteroptera were more sensitive to salinity than the other ten macroinvertebrate groups (Dunlop et al., 2008). In another study, water bug sensitivity to salinity was moderate in seven groups (Kefford et al., 2012). These data suggest that it might be worth investigating whether Nepomorpha is a better predictor of water salinisation than other macroinvertebrate groups.

Summing up, it can be argued that water bugs, particularly corixids, are suitable for water salinity biomonitoring purposes because the tolerance limits of many species differ significantly. Moreover, in many cases, the physiological background of osmoregulation is known. In addition, species richness is negatively correlated with salinity, and LC_50_ values indicated that water bugs might be more sensitive to salinity than other macroinvertebrates. Furthermore, Savage’s method already exists, and its further development and application for biomonitoring seem to be apparently beneficial.

### Anthropogenic light pollution

Many aquatic bug species show positive phototaxis, explaining why light is an ecological trap. This impact has led to the local extinction of the endangered water bug *Lethocerus deyrolli* (Yoon et al., 2010). In addition, numerous flying water bugs have long been known to orient themselves using horizontally polarised light (Schwind, 1983). This ability evolved probably because the water’s surface reflects light in a polarised way. Nowadays, several artificial surfaces appear in nature (oil surfaces, roads, solar panel rooftop or power systems, cars, black plastic sheets etc.) that polarise light and mean “polarised light pollution”(Horváth et al., 2014). Positive polarotaxis is the attractive effect of horizontally polarised light. This phenomenon has been shown in several Corixidae, Belostoma, Notonecta, Nepidae, Gerris and Velia species (Csabai et al., 2006). The sensitivity of the species to light is different. Therefore, it is necessary to discover how sensitive threatened species are too light.

Boda et al. (2014) showed that phototaxis and polarotaxis act synergistically on six Corixidae species in the night, especially on *Sigara falleni* and *Hesperocorixa linnaei*. The impact of light pollution on populations can be significant. According to Bernáth et al. (2008), one ton of aquatic insects (including water bugs) can die per day in summer on a 10 ha on polarised light reflecting black plastic sheet surface. Similar surfaces (e.g. solar farms) are increasing in size and distribution worldwide. Owens et al. (2020) suggest that light pollution is an apparent reason for insect decline. These data show that light pollution can significantly affect the density and dynamics of some aquatic and semi-aquatic bug populations. As there are gaps in data, but the risk of light pollution is significant, this issue should receive future focus.

### How do interspecific interactions influence the effects of pollutants?

The impact of a pollutant cannot be fully understood by looking only at its direct effects, and pollutant assessment will be biased if indirect effects are ignored (Gergs et al., 2013). In many cases, significant indirect effects have been identified. For example, a low concentration of the insecticide fenvalerate inhibited the mosquito *Culex pipiens molestus* alarm responses, and the *Notonecta glauca* predation rate was enhanced (Reynaldi et al., 2011). Multiple effects of endosulfan and a predator’s presence were detected on five water boatmen species (Trekels et al., 2011b). Results showed that whereas the pesticide application and predator cues (visual and chemical) had a synergistic effect on *Sigara lateralis* mortality and *S. iactans* growth rate, an antagonistic effect on the growth rate of *Hesperocorixa linnei* and female *Sigara striata* was observed. All these effects influence the sensitivity rank of these closely related corixid species. However, no evidence was found in a similar experiment that molecular biomarkers (acetylcholinesterase, phenoloxidase, catalase, superoxide dismutase) respond unequivocally to endosulfan and fish (*Gasterosteus aculeatus*) cue effects (Trekels et al., 2012a).

Often difficult to detect is whether observed changes in water bug species density are due to the direct effect of a pesticide or are results of indirect effects as, e.g. through trophic cascades (Relyea, 2005), migration (Bayona et al., 2015; Takahashi et al., 2007), prey density (Cochard et al., 2014) and habitat alteration (Hashimoto et al., 2019). Density changes in field experiments are followed for weeks or months. During such a time frame, density may change oppositely or without a visible pattern, not because the pesticide effect is lacking, but because it will be masked by other environmental effects (Hayasaka et al., 2012; Kobashi et al., 2017).

These studies stress the importance of examining pesticide effects in complex contexts to discover the mechanisms that determine the pesticide effects in the natural environment.

### How do pollutants influence the outcome of interspecific interactions?

There were some cases where interactions between species were not affected by pollutants. *Daphnia magna* and *Notonecta* sp. interactions were not influenced by cadmium in a rockpool food web study (Koivisto et al., 1997). Malathion did not change water bugs’ capture efficiency (Relyea & Hoverman, 2008). Sublethal cadmium exposure to *Chironomus riparius*, *Cloeon dipterum* and *Asellus aquaticus* did not affect the *Notonecta glauca* prey preferences (Brooks et al., 2009).

However, it was more common that pollutants significantly impacted interspecific relationships. Several studies have revealed that the predation efficiency of certain predator species is altered in the presence of pesticides. Relyea and Hoverman (2008) showed that *Belostoma flumineum* predation on tadpoles decreased with increased malathion concentration, most probably because of decreased prey activity and decreased capture ability of the water bug. Sublethal deltamethrin application decreased the predatory efficiency of *Belostoma anurum* significantly on *Aedes aegypti* larvae (Valbon et al., 2018). The predatory efficiency of *Buenoa* sp. on the mosquito *Culex pipiens* larvae was higher if the insecticide Bactimos (Bti) was present in recommended dosage (Rebollartellez et al., 1994). A similar effect was found by Op de Beeck et al. (2016) when *Notonecta maculata* kairomones and Vectobac WG (Bti) were used in the experiment.

A special case is when the insecticide resistance of the prey affects the predatory efficiency of water bugs. For example, significantly more resistant *C. pipiens* larvae were consumed by *Plea minutissima* and *Hyrdometra stagnorum* but not *Sigara lateralis* than susceptible ones (Berticat et al., 2004). Furthermore, *P. minutissima* consumed more resistant than susceptible Bti-exposed *Culex quinquefasciatus* larvae (Delnat et al., 2019). These data show that the general cost of the resistance mechanism reduces the energy available for other life processes, making resistant individuals more accessible to predators. However, Valbon et al. (2019) revealed that *Belostoma anurum* spent more time capturing pyrethroid-resistant than susceptible *Aedes aegypti* larvae because of the anti-predatory strategy of the resistant larvae. This finding disagrees with the previously mentioned results.

There are other data on the effects of pollutants on species interactions. The synergistic effect of nonylphenol and *Notonecta maculata* on *Daphnia magna* was detected, showing a drastic density reduction of the daphnid (Gergs et al., 2013). Endosulfan in low concentration was less lethal to Bullfrog (*Lithobates catesbeianus*) tadpoles in the presence of *Belostoma flumineum* than without it (Hanlon & Relyea, 2013).

Studying the impact of pollutants on interspecific effects is a relatively new research area. That is why little is known about, e.g. the top-down and life-history changes associated with changes in predation efficacy in a polluted environment.

### The problem of species sensitivity differences

This issue is interesting if we aim to find a surrogate species for laboratory or field tests. The species-specific sensitivity differences of aquatic insects to insecticides are detected in several cases (Anderson, 1989). Such a review has not yet been performed for water bugs.

**Fig. 6:**
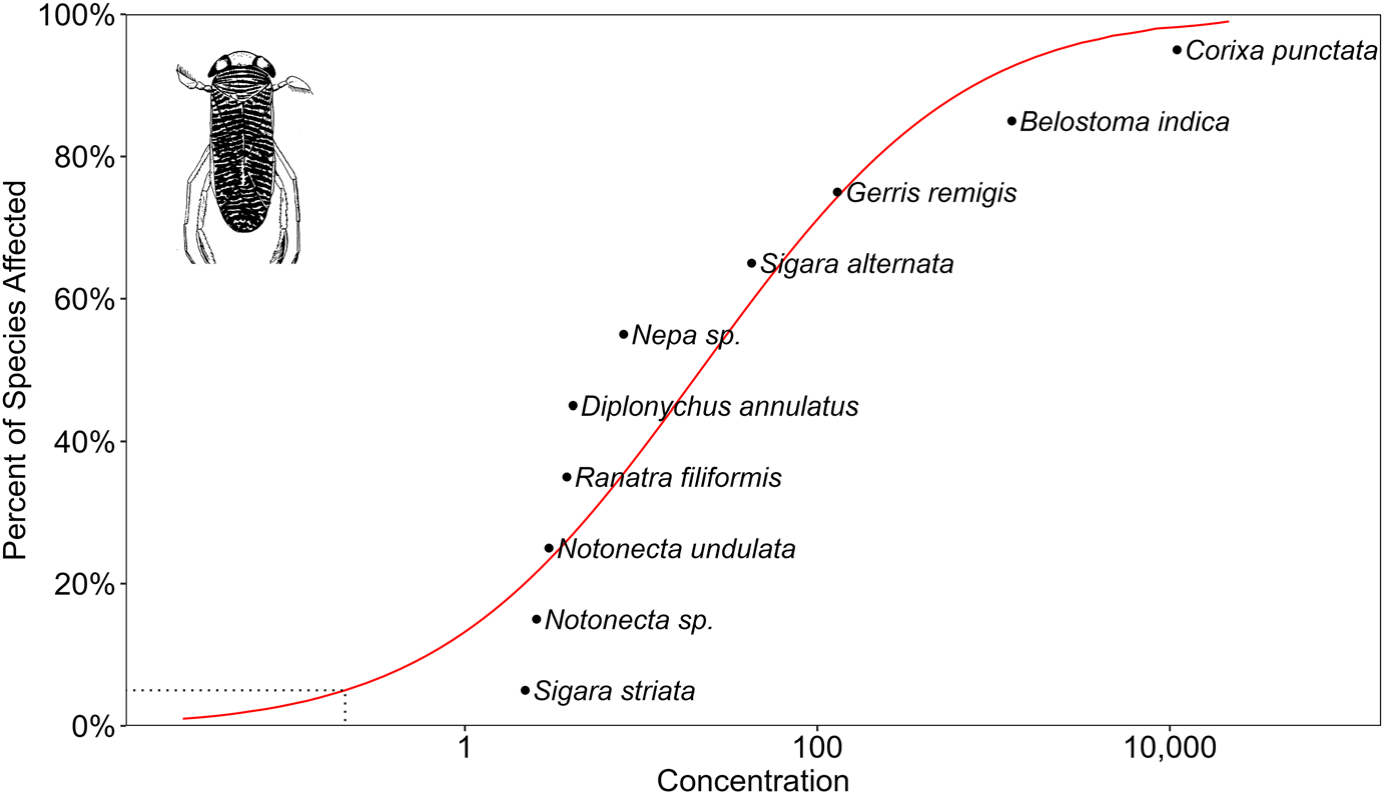
SSD curve for 28- to 96-h LC_50_ data for all organochloride pesticides, tested with different water bug species

**Fig. 7:**
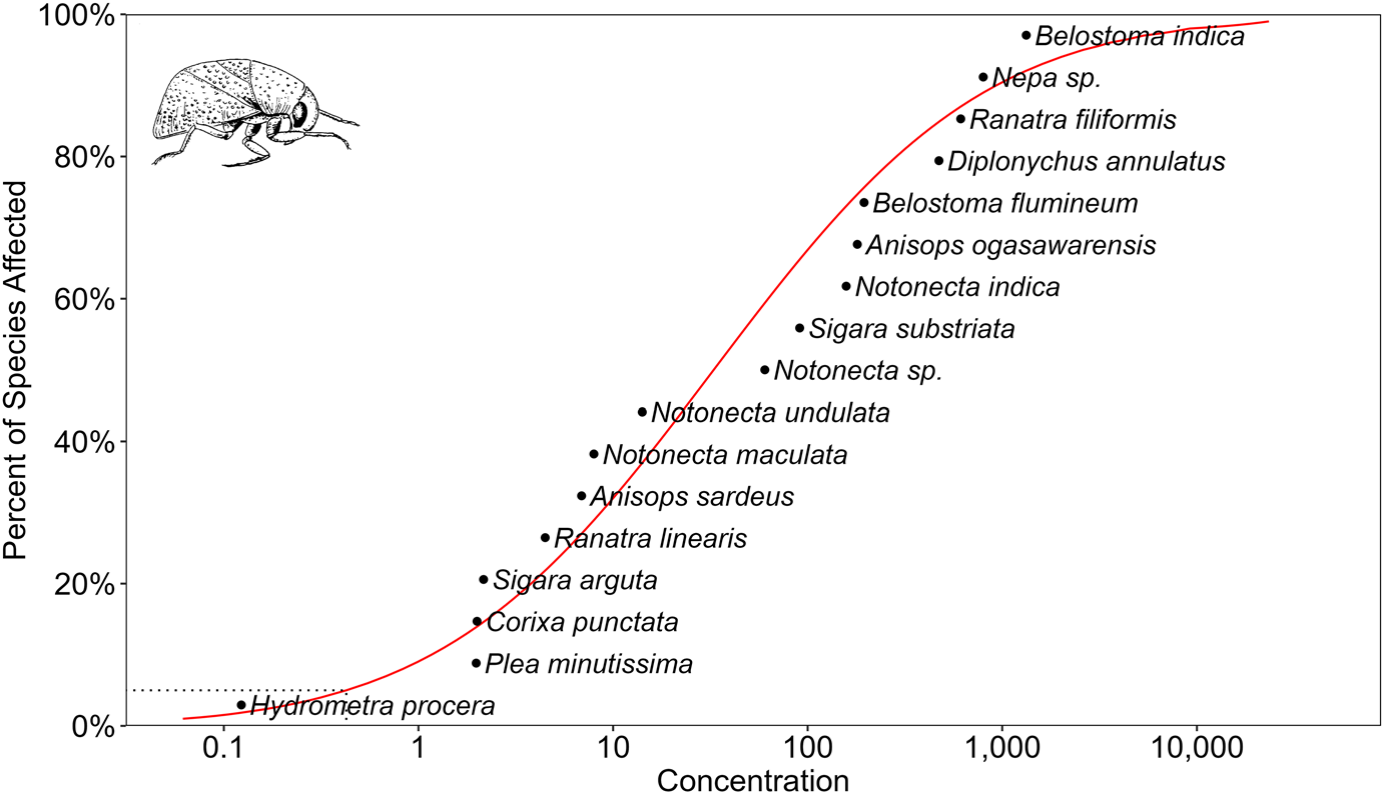
SSD curve for 28- to 96-h LC_50_ data for all organophosphate pesticides, tested with different water bug species

**Fig. 8:**
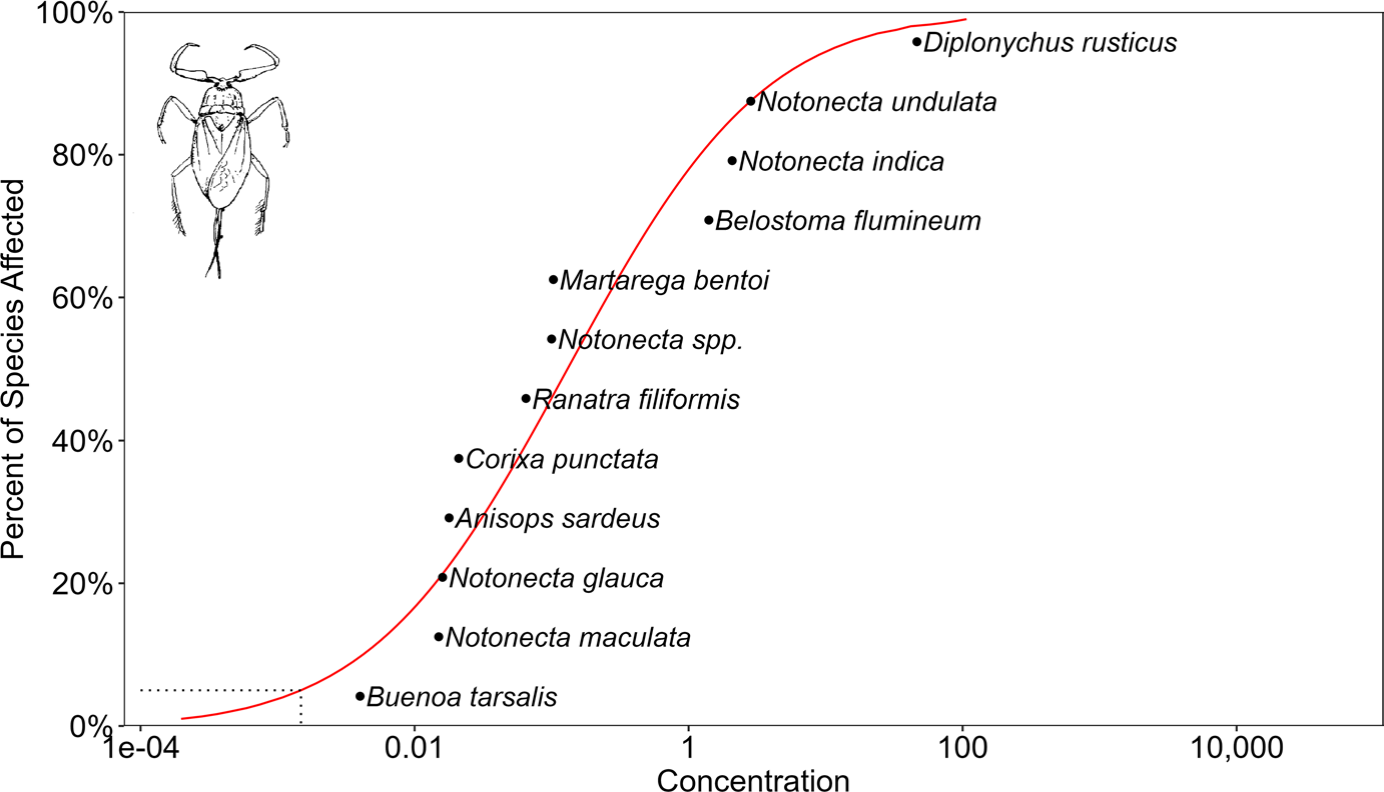
SSD curve for 28- to 96-h LC_50_ data for all pyrethroid pesticides, tested with different water bug species

Our datasets for chemical pesticides and pesticides derived from microorganisms indicates (Tables S1–S3) that although there are comparable data on species’ sensitivity (Konar, 1969; Miles et al., 2017), these cannot be generalised because the number of repeated experiments is very low. So, currently, it is impossible to make general statements about species’ sensitivity for a given active ingredient or formulation. Our results of the species sensitivity distribution (SSD) analysis also reinforce and quantify the well-known fact that water bugs are more sensitive to pyrethroids than organophosphates and organochlorides (Figs. 6, 7, and 8). According to the HC_5_ values, the toxicity ranking of the pesticides was as follows: pyrethroids (0.0017 µg/L), > organophosphates (0.235 µg/L) > organochlorides (0.257 µg/L). It is also unambiguous that species sensitivity depends on the insecticide class. For example, *Corixa punctata* is relatively insensitive to organochlorides, moderately sensitive to pyrethroids and relatively sensitive to organophosphates. The relative position of the species to each other consequently differ according to the pesticide classes, and no regularity in sensitivity can be noticed. Furthermore, there is no indication that species sensitivity values in the same genus are closer to each other than species sensitivity values with species belonging to different genera (Figs. 5, 6, and 7). However, this feature may change if more data will be gained and examined from a phylogenetic perspective, too (Hammond et al., 2012).

We could test the relative sensitivity of two species on a relatively large dataset. Several dose–response experiments were performed on *Anisops bouvieri* and *Diplonychus indicus* with roughly similar methods searching the effects of green pesticides (chemicals of phytogenic origin and silver nanomaterial synthesised by using plant extracts). Data in Table S4 shows that *A. bouvieri* proved to be more sensitive than *D. indicus* in 44 out of 48 experiments against 45 different green pesticides. Based on these data, a significantly lower mean LC_50_ value could be calculated for *A. bouvieri* (paired *t*-test; *t* =  −5.3, *p* < 0.001) even though a wide range of pesticide types were applied and the LC_50_ values varied between a broad range. This result demonstrates that there can be stable differences in sensitivity between different species to several pollutants. Consequently, it is possible that stable species differences have not been found in other cases because there are not enough replicate studies available.

Based on dose–response field experiments on communities, Daam et al. (2009) showed that *Corixida* spp. are most susceptible to carbendazim than the Notonectida species. *Ranatra linearis*, *Gerris* sp. and *Notonecta* spp. proved to be more sensitive to imidacloprid than other macroinvertebrates except for *Cloeon* sp. (Sumon et al., 2018). The problem with the results of these experiments is that in lack of species-level identification, they are not comparable with other studies.

## Future directions

There is a need for simple, sensitive, cheap and stable early warning methods for water pollution biomonitoring systems. Several new ecotoxicological research areas have recently emerged. The results of these will likely be used in the biomonitoring of water bugs in the future. Four such areas are discussed below. These are the study and use of pollution-induced (1) molecular, (2) histopathological, (3) morphological and (4) behavioural end-points.

An increasing number of data about molecular level changes are achievable due to increasing environmental pollution and with the development of the “omics” methodologies in ecotoxicology. Biochemical biomarkers for water bugs are also under intense development. Boonthai et al. (2000) suggested acetylcholinesterase activity in water insects (including *Sigara argute*) as a possible marker of chlorpyrifos and atrazine pollution of waters. There are situations where pollutants increase the activity of molecular markers in water bugs. Endocrine disruptors have a substantial effect on the vitellogenin production of *Lethocerus deyrollei*. The application of 17β-estradiol and bisphenol A enhanced, but 4-t-octylphenol and 4-nonylphenol decreased the vitellogenin expression disrupting the reproductive physiology processes (Nagaba et al., 2011). A detailed study with *Belostoma elegans* found that lipid metabolism and increased antioxidant enzyme activity (superoxide dismutase, catalase) are suitable biomarkers of organic matter pollution, and sex-specific differences were also found in lactate concentration (Lavarias et al., 2017). The glutathione S-transferase activity of *Cylindrostethus palmaris* was higher in oil palm plantations than in forest streams (Mendes et al., 2020). As molecular methods become more straightforward and cheaper, they will become more widespread in practice.

In other studies, the activity of molecular markers was reduced. Endosulfan inhibited four enzyme activities (acetylcholinesterase, catalase, phenoloxidase, superoxide dismutase) in four corixid species belonging to three different genera (Trekels et al., 2012a). Reduced cytochrome P450 monooxygenases activity was found in *Belostoma anurum* nymphs when sublethally exposed to pyriproxyfen (Valbon et al., 2021). From the data accumulated so far, it appears that the molecular data are context-dependent. Both contaminant and experimental type setup influence the enzyme activity level.

Histopathological end-points have been successfully used in different vertebrate and invertebrate toxicity studies, but few studies have been conducted on water bugs. Histopathological studies proved apparent differences in some organs collected from clean and polluted water. For example, it was found that Malpighian tubule cells of *Lethocerus niloticum* living in water contaminated with domestic pollutants, heavy industry waste and agricultural run-off had pleomorphic mitochondria, irregular laminated concretions, increased number of lysosomes and cytoplasm lysis (Sorour, 2001). Histological deviations in the reproductive system, e.g. aggregated clumps of heterochromatin, disorganised mitochondria and nucleolus of *Anisops sardeus* and *Sphaerodema urinator*, and the seminal vesicle of *Laccotrephes ruber* had highly disintegrated epithelial cells in specimens collected from heavy metal polluted waters (Kheirallah, 2015). In our opinion, it would be worthy of performing more extensive histopathological studies on water bugs because most species are easy to collect, relatively large, so tissues are easy to dissect, and the cost of studies is low.

Morphological disorders due to pollutants were detected in some cases. Aberrations were induced by an insect growth regulator (fenoxycarb) in *Notonecta unifasciata* (Miura & Takahashi, 1987). The methoprene S disturbed normal metamorphosis of the last larval instar of *Ilyocoris cimicoides* (Gelbic et al., 1994). Abnormalities occurred at the nymph-adult ecdysis in both cases. Tooby and Macey (1977) found that the herbicide dichlobenil inhibited pigmentation of *Corixa punctata* and *Sigara dorsalis* fifth instar nymphs and adults. Despite these results, morphological biomarker research is an underdeveloped field of water bug ecotoxicology, although morphologically deformed specimens could certainly be collected from contaminated waters, and the change is easily detectable.

Behavioural ecotoxicology is a rapidly evolving field of science, but few such studies have been performed on water bugs. Deltamethrin markedly influenced the swimming behaviour of *Buenoa tarsalis* in a short-term experiment (Gutiérrez et al., 2017b). The further important point was that the behaviour of *B. tarsalis*, and the relatively closely related species from an ecological point of view, *Martarega bentoi* (both are backswimmers), differed significantly if the same insecticide concentration was applied. A single pulse application of endosulfan decreased the swimming speed of *Sigara iactans* as well as the total activity of cholinesterase in the body (Trekels et al., 2012b). Low doses of carbon and silver nanoparticles did not reduce *Lethocerus indicus* predation efficiency (Murugan et al., 2016), but increased salinity level decreased it in laboratory studies (Chandramohan et al., 2008).

A new, emerging field is freshwater ecoacoustics, including the study of aquatic noise pollution (Kunc et al., 2016). It has long been well known that many water bug species, especially corixids, produce sound as a component of mating behaviour (Aiken, 1985), so noise pollution is a potential threat to reproductive success. Van der Lee et al. (2020) found a close relation between specific acoustic indices based on the sound production of the invertebrate community (including water bugs) and dissolved oxygen concentrations. Although no relationship has been found between water quality and the indices tested, the method is considered suitable for monitoring purposes after further refinement. Similarly, Desjonquères et al. (2018) developed a method for monitoring the acoustic activity of *Micronecta scholtzi*, which could be used to detect anthropogenic effects.

Linking molecular, histopathological, morphological or behavioural biomarkers with ecotoxicological events is a promising but still largely unexplored field. More analyses are necessary to validate the abovementioned biomarkers, with particular regard to the experimental setups, background mechanisms and differences between species in distinct geographical areas.

## Suggestions

Our review shows that several studies on pollution effects on water bugs are available. These include comprehensive laboratory, semi-field and field studies. Thus, there is no reason to believe that the available databases are insufficient to incorporate water bugs in some areas of water pollution biomonitoring.

Aquatic and semi-aquatic Heteroptera are regarded as relative insensitive taxa to most insecticides (Liess & Ohe, 2005; Rico & Van den Brink, 2015; Rubach et al., 2010). However, such a generalisation is misleading because LC_50_ values may differ with one order of magnitude or more, in replicated studies, with the same pesticide and species, as seen, e.g. in Ernst et al. (1991). Thus, only comparative studies with the same pesticide and methodology can accurately determine the sensitivity of aquatic and semi-aquatic bugs to pesticides. Rubach et al. (2010) and Rico and Van den Brink (2015) suggest that relative species sensitivity is a more or less stable phenomenon, at least regarding one insecticide class. We confirm this claim for two water bug species, as we found that a broad spectrum of plant-derived pesticides are more toxic on *Anisops bouvieri* than *Diplonychus indicus*.

In many cases, interactions significantly influence the effects of pesticides. In the future, more attention should be paid to the combined effects of chemicals used commonly together (pesticides, fertilisers etc.) and other organisms in the habitat. Reactions under different abiotic circumstances (temperature, pH etc.) are also neglected research areas.

Water quality is often assessed by pollution indices. For example, according to the tolerance scores of macroinvertebrates in Flanders, Aphelocheirus is a pollution sensitive taxon, but all others (21 water bug genus) are labelled as moderately sensitive by Gabriels et al. (2010). Similarly, *Velia* sp., *Ranatra* sp. and *Hydrometra longicapitis* were moderately tolerant taxons to pollution in a polluted river in Malaysia (Al-Shami et al., 2011). This relative insensitivity to water pollution compared to the EPT group may be the reason why water bugs are neglected in biomonitoring systems. This statement may be true for pesticide effects on the water bug species, but there are some special issues, as presented below.

There is a valuable amount of data about the ecotoxicological effects of Cd and Hg on water bugs. Cd and Hg concentrations of the Heteroptera species can vary by four orders of magnitude in field populations. As biomagnification is observed in predatory water bugs, they are candidates for monitoring Hg contamination.

Based on all data, certain *Halobates* spp. and *Gerris* spp. can be suggested for Cd sentinels (Beeby, 2001). *Halobates* spp. (*H. sobrinus*, *H. micans*, *H. sericeus*) proved to be useful sentinels for Cd pollution of the oceans. For Halobates, there is sufficient information on the differences in the heavy metal content of the species and the environmental origin of Cd. *Gerris* spp. (*G. argentatus*, *G. lateralis*, *G. odontogaster*, *G. thoracicus*) seem to be suitable as Cd sentinels. Many promising data are available, showing a high concentration of Cd in the bodies of different Gerris species in the field. There is uncertainty about how much of the Gerris’ food originates from water and from land. Therefore, it is necessary to study in detail the heavy metal ecotoxicology of *Gerris* spp. in the future.

We suggest involving water bugs in eutrophication and salinisation biomonitoring. Micronecta species composition in particular, but to some extent, the species composition of Corixidae seems to be suitable for eutrophication level identification. There is information about different salinity tolerance in several species, primarily corixids. Therefore, the available dataset may establish a corixid species scoring study as community analysis, and a further development of the Savage’s method. Furthermore, species richness was found negatively correlated with salinisation. That is why this community parameter is suggested to be involved in water pollution biomonitoring. Finally, Corixidae is suggested as an indicator of nitrate level (Collins & Fahrig, 2020).

## Supplementary Information

Below is the link to the electronic supplementary material.

Supplementary file1 (PDF 943 KB)
