# Supplementary material for: Pollution impacts on water bugs (Nepomorpha, Gerromorpha): state of the art and their biomonitoring potential
Source: Environ Monit Assess. 2022 Mar 28;194(4):301. doi: 10.1007/s10661-022-09961-2 (PMC8960648; doi:10.1007/s10661-022-09961-2)
Supplement: Supplementary file 1 — Supplementary file1 (PDF 943 KB) [file 10661_2022_9961_MOESM1_ESM.pdf]

Pollution impacts on water bugs (Nepomorpha, Gerromorpha): state-of-the-art and their biomonitoring potential

Gábor Bakonyi
Tamás Vásárhelyi
Borbála Szabó

G. Bakonyi
Department of Zoology and Ecology, Hungarian University of Agriculture and Life Sciences, 2100 Gödöllő, Hungary
e-mail: Bakonyi.Gabor@mkk.szie.hu
ORCID number: 0000-0002-7132-7970

T. Vásárhelyi
1125 Budapest, Diós árok 16/II., Hungary

B. Szabó
Centre for Ecological Research, Institute of Ecology and Botany, “Lendület” Landscape and Conservation Ecology Research Group, 2163 Vácrátót, Hungary
ORCID number: 0000-0001-7587-1597

Supplementary Information

Table S1. Summary of the insecticide effects on water-bug species (laboratory studies).

species	pesticide		LC50 (µg/L)				EC50 immobilisation (µg/L)			reference
	commercial name	active ingredient	24 h	48 h	96 h	168 h	24 h	48 h	96 h	
Notonecta undulata	Abate	temephos	6.2	5.0						Fales et al., 1968
Notonecta undulata	S.B. Penick 1382	resmethrin	2.5	2.8						Mills et al., 1969
Notonecta undulata	S.B. Penick 1390	resmethrin	2.4	2.4						Mills et al., 1969
Notonecta undulata	Abate	temephos		5.4						Mills et al., 1969
Notonecta sp.	Nuvan 100 EC	DDVP				1.23				Konar, 1969
Diplonychus annulatus	Nuvan 100 EC	DDVP				85.0				Konar, 1969
Ranatra filiformis	Nuvan 100 EC	DDVP				130.0				Konar, 1969
Nepa sp.	Nuvan 100 EC	DDVP				269.0				Konar, 1969
Belostoma indica	Nuvan 100 EC	DDVP				280.0				Konar, 1969
Notonecta sp.	Dimecron 100	phosphamidon				2950.0				Konar, 1969
Diplonychus annulatus	Dimecron 101	phosphamidon				2630.0				Konar, 1969
Ranatra filiformis	Dimecron 102	phosphamidon				2880.0				Konar, 1969
Nepa sp.	Dimecron 103	phosphamidon				2370.0				Konar, 1969
Belostoma indica	Dimecron 104	phosphamidon				6300.0				Konar, 1969
Ranatra filiformis		heptachlor				3.79				Konar, 1970
Notonecta sp.		heptachlor				2.55				Konar, 1970
Diplonychus annulatus		heptachlor				4.11				Konar, 1970
Nepa sp.		heptachlor				7.96				Konar, 1970
Belostoma indica		heptachlor				1270.0				Konar, 1970
Notonecta undulata		chlorpyrifos	35.2							Roberts et al., 1973
Gerris remigis		mirex	130.0 (20.0-39.0)							Naqvi and de la Cruz, 1973

Corisella decolor	methoprene			1650	Miura and Takahashi, 1974
Notonecta unifasciata	methoprene	1200			Miura and Takahashi, 1974
Notonecta sp.	2-(digeranylamino)-ethanol	4900 (3980-6030)			Marking, 1974
Notonecta undulata	dieldrin			1.0	Federle and Collins, 1976
Notonecta undulata	lindane		7.0	3.0	Federle and Collins, 1976
Notonecta undulata	DDT	70.0	20.0	20.0	Federle and Collins, 1976
Notonecta undulata	parathion	20.0	10.0		Federle and Collins, 1976
Notonecta undulata	malathion	720.0	110.0	80.0	Federle and Collins, 1976
Notonecta undulata	dichlorvos		60.0	20.0	Federle and Collins, 1976
Notonecta undulata	carbaryl		360.0	200.0	Federle and Collins, 1976
Notonecta undulata	propoxur		300.0	160.0	Federle and Collins, 1976
Notonecta sp.	formalin	4500 (3006-6735)		835 (652-1069)	Bills et al., 1977
Corixa punctata	cypermethrin	> 5.0			Stephenson, 1982
Notonecta indica	permethrin	2.8 (1.8-4.0)			Alexander et al., 1982
Notonecta indica	temephos	54.0 (44.0-67.0)			Alexander et al., 1982
Notonecta indica	ethyl parathion	141.0 (117.0-179.0)			Alexander et al., 1982

Notonecta indica		fenthion	516.0 (422.0-634.0)							Alexander et al., 1982
Corixa punctata		pentachlorophenol		11 000.0						Sloof, 1983
Sigara alternata	Thiodan 50 WP	endosulfan	13.0							Ernst et al., 1991
Sigara alternata	Thiodan 50 WP	endosulfan	75.0 (27.0-123.0)							Ernst et al., 1991
Sigara alternata	Thiodan 50 WP	endosulfan	269.0 (59.0-479.0)							Ernst et al., 1991
Sigara alternata	Thiodan 50 WP	endosulfan	> 50.0	12.3 (0.9-50.0)			28			Ernst et al., 1991
Corixa punctata	Dursban 4E	chlorpyrifos		6.0 (4.2-8.5)	2.0 (1.5-2.6)			3.2 (2.4-4.3)	1.7 (1.1-2.5)	van Wijngaarden et al., 1993
Corixa sp.		lambda-cyhalothrin						0.03		Maund et al., 1998
Notonecta spp.		cypermethrin		0.1						Sherratt et al., 1999
Sigara arguta		chlorpyrifos		2.16 (1.38-3.28)						Boonthai et al., 2000
Sigara arguta		atrazine		29.04 (16.67-55.26)						Boonthai et al., 2000
Anisops sardeus	Sumithion	fenitrothion	16.7 (14.9-20.8)	8.61 (7.8-9.3)						Lahr et al., 2001
Anisops sardeus	Dursban	chlorpyrifos	1.58 (1.53-1.62)	0.90 (0.88-0.92)						Lahr et al., 2001
Anisops sardeus	Fyfanon	malathion	70.7 (57.4-78.0)	42.2 (40.5-44.9)						Lahr et al., 2001

Anisops sardeus	Ficam	bendiocarb	389 (287-567)	373 (275-567)						Lahr et al., 2001
Anisops sardeus	Volaton+Unden	propoxur+phoxim	3.38 (2.65-4.52)	1.91 (1.75-2.08)						Lahr et al., 2001
Anisops sardeus	Decis	deltamethrin	0.013 (0.011-0.014)	0.012 (0.010-0.014)						Lahr et al., 2001
Anisops sardeus	Karate	lambda-cyhalothrin	0.026 (0.024-0.031)	0.025 (0.023-0.031)						Lahr et al., 2001
Anisops sardeus	Bulldock	betacyfluthrin	0.021 (0.016-0.026)	0.019 (0.015-0.025)						Lahr et al., 2001
Anisops sardeus	Dimilin	diflubenzuron	2123 (1960-2210)	1937 (1800-2020)						Lahr et al., 2001
Anisops sardeus	Nomolt	teflubenzuron	249 (233-267)	249 (233-267)						Lahr et al., 2001
Anisops sardeus	Alsystin	triflumuron	199 (168-250)	189 (168-228)						Lahr et al., 2001
Cymatia coleoptrata		esfenvalerate							3.3 µg/L	Samsoe-Petersen et al., 2001
Notonecta glauca	Karate	lambda-cyhalothrin		0.023 (0.013–0.04)	0.016 (0.008–0.036)			0.015 (0.01–0.022)	0.016 (0.008–0.036)	Schroer et al., 2004
Ranatra filiformis		cypermethrin	0.12 (0.11–0.13)	0.09 (0.08–0.10)	0.065 (0.06–0.07)					Saha and Kaviraj, 2008
Notonecta maculata		gamma-cyhalothrin	>0.361	0.066 (0.042–0.102)	0.015 (0.011–0.022)		0.013	0.006	0.005 (0.004–0.006)	van Wijngaarden et al., 2009

Corixa punctata		gamma-cyhalothrin	>0.361	0.065 (0.037–0.114)	0.021 (0.012–0.038)		0.013 (0.010–0.018)	0.012 (0.011–0.014)	0.012 (0.011–0.014)	van Wijngaarden et al., 2009
Diplonychus rusticus	Excel Endohyper	endosulfan and cypermethrin	140.6 (105.0-188.3)	113.8 (109.8-118.0)	91.8 (89.5-94.2)					Kalimuthu and Selvaraj Pandian 2010
Notonecta maculata		chlorpyrifos	>16	23.9 (15.0–38.2)	7.97 (5.34–11.9)		19.5 (12–31.8)	9.07 (7.18–11.5)	2.78	Rubach et al., 2011
Plea minutissima		chlorpyrifos	11.2 (8.95–13.93)	5.94 (4.026–8.765)	1.98 (1.32–2.99)		5.35 (4.19–6.83)	2.65 (2.06–3.39)	1.29 (0.92–1.8)	Rubach et al., 2011
Ranatra linearis		chlorpyrifos	22.5 (15.3–33.2)	11.97 (NC)	4.48 (2.39–8.41)		19.9 (13.3–29.9	12 (NC)	3.33 (2.95–3.76)	Rubach et al., 2011
Plea minutissima		imidacloprid			37.5				35.9 (31.1–41.5)	Roessink et al., 2013
Notonecta spp.		imidacloprid			> 10000				18.2 (9.24–35.7)	Roessink et al., 2013
Micronecta spp.		imidacloprid			28.2 (17.6–45.2)				10.8 (9.72–12.0)	Roessink et al., 2013
Belostoma flumineum		esfenvalerate			1.62 (0.88–3.00)					Halstead et al., 2015
Belostoma flumineum		lambda-cyhalothrin			0.25 (0.14–0.43)					Halstead et al., 2015
Belostoma flumineum		permethrin			6.85 (2.64–17.8)					Halstead et al., 2015
Belostoma flumineum		chlorpyrifos			37.0 (24.3–56.5)					Halstead et al., 2015

Belostoma flumineum		malathion			2695.0 (1544.0–7558.0)		Halstead et al., 2015
Belostoma flumineum		terbufos			74.3 (18.8–117)		Halstead et al., 2015
Notonecta undulata		allethrin		29.0			Antwi and Reddy, 2015
Notonecta undulata		dimethrin		0.1			Antwi and Reddy, 2015
Notonecta undulata		tetramethrin		33.8			Antwi and Reddy, 2015
Notonecta undulata		resmethrin		1.9			Antwi and Reddy, 2015
Notonecta undulata		bioresmethrin		1.2			Antwi and Reddy, 2015
Plea minutissima		imidacloprid				189	van den Brink et al., 2016
Belostoma flumineum		clothianidin		79 (52-107)			Miles et al., 2017
Notonecta undulata		clothianidin		59 (35-107)			Miles et al., 2017
Hesperocorixa atopodonta		clothianidin		56 (39-82)			Miles et al., 2017
Buenoa tarsalis	Decis 25EC	deltamethrin	0.004 (0.003 – 0.006)				Gutiérrez et al., 2017
Martarega bentoi	Decis 25EC	deltamethrin	0.103 (0.039 - 0.228)				Gutiérrez et al., 2017
Diplonychus rusticus	Temper EC	lambda-cyhalothrin	50 (1-90)				Reegan et al., 2020
Diplonychus rusticus	Superkiller 10%EC	temephos	100 (10-290)				Reegan et al., 2020
Diplonychus rusticus	Baton	cypermethrin	20 (1-07)				Reegan et al., 2020

Hydrometra procera		diazinon	0.123 (0.066–0.646)			Murata and Tanaka, 2020
Hydrometra procera		fenobucarb	0.027 (0.011–0.049)			Murata and Tanaka, 2020
Hydrometra procera		cartap		0.094 (0.025–0.318)		Murata and Tanaka, 2020
Buenoa sp.	Dimilin	diflubenzuron			2770	Ferreira et al., 2020

Table S2a. Summary of the insecticide effects on water-bug species (semi-field and field studies).

species	pollutant		applied rate	effect on density	remarks	author
	commercial name	active ingredient				
Trichocorixa sp., Notonecta sp.	Monsanto 0585	(2,6-di-t, butyl-4-(a,a, dimethyl benzyl) phenol)	3.36 (kg/ha)	no effect	mesocosmos	Steelman and Schilling, 1972
Corisella decolor, Notonecta unifasciata		methoprene	100 μg/L	no effect	mesocosmos	Miura and Takahashi, 1973
Notonecta spp., Corixidae	TH-6040		0.001-0.28 (g/ha), six concentrations	no effect	field	Steelman et al., 1975
Notonecta spp., Corixidae	R-20458		0.001-0.28 (g/ha), six concentrations	no effect	field	Steelman et al., 1975
Notonecta spp., Corixidae	Altosid		0.001-0.28 (g/ha), six concentrations	no effect	field	Steelman et al., 1975
Notonecta spp., Corixidae	Monsanto 0585		0.001-0.28 (g/ha), six concentrations	no effect	field	Steelman et al., 1975
Trichocorixa louisianae nymphs, Buenoa spp. nymphs		diflubenzuron	0.028 (a.i. g/ha) (6 times)	significant decrease	field	Farlow et al., 1978
Mesovelia mulsanti, Trichocorixa louisianae		diflubenzuron	0.028 (a.i. g/ha) (6 times)	significant increase	field	Farlow et al., 1978
Buenoa sp. adult, Belostoma sp nimph and adult.		diflubenzuron	0.028 (a.i. g/ha) (6 times)	no effect	field	Farlow et al., 1978
Belostoma		methoprene	110 (a.i. g/ha)	significant decrease	field	Case and Washino, 1978
Notonectidae		cypermethrin	70 (a.i. g/ha)	no effect	field	Crossland et al., 1982
Corixidae		cypermethrin	71 (a.i. g/ha)	no effect	field	Crossland et al., 1982
Corisella spp.	ISA-20E	liquid isostearyl alcohol	0.25, 0.5, 1.0 ml/m^2	decrease	field	Takahashi et al., 1984
Notonecta unifasciata	ISA-20E	liquid isostearyl alcohol	0.25, 0.5, 1.0 ml/m^3	decrease	field	Takahashi et al., 1984

Corixidae	Ripcord	cypermethrin	25 (a.i. g/ha)	decrease	field	Shieres and Bennett 1985
Corisella decolor		triphenyltin hydroxide	1160 (a.i. g/ha)	significant decrease	field	Grigarick et al., 1990
Merragata hebroides		triphenyltin hydroxide	1160 (a.i. g/ha)	significant decrease	field	Grigarick et al., 1990
Corisella decolor		triflumuron	280 (a.i. g/ha)	significant increase	field	Grigarick et al., 1990
Corisella decolor		triflumuron	420 (a.i. g/ha)	significant increase	field	Grigarick et al., 1990
Corisella decolor		diflubenzuron	280 (a.i. g/ha)	significant increase	field	Grigarick et al., 1990
Notonecta undulata		triflumuron	280 (a.i. g/ha)	significant decrease	field	Grigarick et al., 1990
Notonecta undulata		triflumuron	420 (a.i. g/ha)	significant decrease	field	Grigarick et al., 1990
Notonecta undulata		diflubenzuron	280 (a.i. g/ha)	significant decrease	field	Grigarick et al., 1990
Corixidae nimphs	Dimilin WP-25	diflubenzuron	70 (a.i. g/ha)	significant decrease	field	Sundaram et al., 1991
Sigara alternata	Bravo 500	chlorothalonil	875 (a.i. g/ha)	decrease	field	Ernst et al. 1991
Corixa punctata		chlorpyrifos	5 (µg/L)	decrease	microcosmos	van Breukelen and Brock, 1993
Notonecta sp.		chlorpyrifos	5 (µg/L)	decrease	microcosmos	van Breukelen and Brock, 1993
Sigara sp.		chlorpyrifos	5 (µg/L)	decrease	microcosmos	van Breukelen and Brock, 1993
Corixa punctata		chlorpyrifos	35 (µg/L)	decrease	microcosmos	van Breukelen and Brock, 1993
Notonecta sp.		chlorpyrifos	35 (µg/L)	decrease	microcosmos	van Breukelen and Brock, 1993
Sigara sp.		chlorpyrifos	35 (µg/L)	decrease	microcosmos	van Breukelen and Brock, 1993
Microvelia horvathi	Applaud	buprofezin	80 (a.i. g/ha)	no effect	field	Kanaoka et al., 1994

Microvelia atrolineata	Applaud	buprofezin	80 (a.i. g/ha)	no effect	field	Kanaoka et al., 1994
Gerris paludum insularis	Applaud	buprofezin	80 (a.i. g/ha)	no effect	field	Kanaoka et al., 1994
Sigara substriata	Applaud	buprofezin	80 (a.i. g/ha)	no effect	field	Kanaoka et al., 1994
Trichocorixa reticulata	Altosid	methoprene S	208 (a.i. g/ha)	no effect	field	Lawler et al., 2000
Anisops sardeus	Sumithion	fenitrothion	500 (a.i. g/ha)	significant decrease	field	Lahr et al., 2000
Anisops sardeus	Dimilin	diflubenzuron	60 (a.i. g/ha)	non consistent	field	Lahr et al., 2000
Anisops sardeus	Decis	deltamethrin	15 (a.i. g/ha)	significant decrease	field	Lahr et al., 2000
Anisops sardeus	Ficam	bendiocarb	100 (a.i. g/ha)	non consistent	field	Lahr et al., 2000
A. debilis perplexus	Sumithion	fenitrothion	500 (a.i. g/ha)	significant decrease	field	Lahr et al., 2000
A. debilis perplexus	Dimilin	diflubenzuron	60 (a.i. g/ha)	non consistent	field	Lahr et al., 2000
A. debilis perplexus	Decis	deltamethrin	15 (a.i. g/ha)	significant decrease	field	Lahr et al., 2000
A. debilis perplexus	Ficam	bendiocarb	100 (a.i. g/ha)	non consistent	field	Lahr et al., 2000
A. varius	Decis	deltamethrin	15 (a.i. g/ha)	significant decrease	field	Lahr et al., 2000
A. varius	Ficam	bendiocarb	100 (a.i. g/ha)	non consistent	field	Lahr et al., 2000
A. spp. nymphs	Sumithion	fenitrothion	500 (a.i. g/ha)	significant decrease	field	Lahr et al., 2000
A. spp. nymphs	Dimilin	diflubenzuron	60 (a.i. g/ha)	non consistent	field	Lahr et al., 2000
A. spp. nymphs	Decis	deltamethrin	15 (a.i. g/ha)	significant decrease	field	Lahr et al., 2000
A. spp. nymphs	Ficam	bendiocarb	100 (a.i. g/ha)	non consistent	field	Lahr et al., 2000
Notonectidae	Noxfish	rotenon	150 (µg/L)	significant decrease	field	Melaas et al. 2001
Pleidae	Noxfish	rotenon	150 (µg/L)	no effect	field	Melaas et al. 2001
Corixidae	Noxfish	rotenon	150 (µg/L)	no effect	field	Melaas et al. 2001

Micronecta sp.		endosulfan	0.13, 0.38, 1.07, 6.14 µg /L	no effect	field	Hose et al., 2002
Notonectidae		endosulfan	0.13, 0.38, 1.07 µg /L	no effect	field	Hose et al., 2002
Notonectidae		endosulfan	6.14 µg /L	significant decrease	field	Hose et al., 2002
Micronecta sp.		endosulfan	1 µg /L	no effect	field	Hose et al., 2003
Micronecta sp.		endosulfan	6.87, 30.7 µg /L	significant decrease	field	Hose et al., 2003
Agraptocorixa sp.		endosulfan	1 µg /L	no effect	field	Hose et al., 2003
Agraptocorixa sp.		endosulfan	6.87, 30.7 µg /L	significant decrease	field	Hose et al., 2003
Notonecta indica	Icon 6.2 FS	fipronil	28 (a.i. g/ha)	adversely affected	semi-field	Dennett et al., 2003
Notonecta indica	Karatez 2.08 CS	lambda-cyhalothrin	33 (a.i. g/ha)	less harmful	semi-field	Dennett et al., 2003
Notonecta undulata	Sevin	carbaryl	0.51 (mg/L)	no effect	mesocosmos	Relyea, 2005
Notonecta undulata		malathion	0.32 (mg/L)	no effect	mesocosmos	Relyea, 2005
Notonecta undulata	Roundup	glyphosate	3.8 (mg/L)	no effect	mesocosmos	Relyea, 2005
Notonecta undulata		2,4-D	0.12 (mg/L)	significant increase	mesocosmos	Relyea, 2005
Belostoma flumineum	Sevin	carbaryl	0.51 (mg/L)	no effect	mesocosmos	Relyea, 2005
Belostoma flumineum		malathion	0.32 (mg/L)	no effect	mesocosmos	Relyea, 2005
Belostoma flumineum	Roundup	glyphosate	3.8 (mg/L)	no effect	mesocosmos	Relyea, 2005
Belostoma flumineum		2,4-D	0.12 (mg/L)	no effect	mesocosmos	Relyea, 2005
Corixidae	Dursban 40 EC	chlorpyrifos	0.1, 1 (a.i. µg/L)	no effect	mesocosmos	Daam et al., 2008
Corixidae	Dursban 40 EC	chlorpyrifos	10, 100 (a.i. µg/L)	significant decrease	mesocosmos	Daam et al., 2008
Plea minutissima	Match	lufenuron	1, 2, 3 (a.i. µg/L)	significant decrease	mesocosmos	Brock et al., 2009
Corixidae	Bavistin FL	carbendazim	3.3, 33, 100, 1000 µg/L	significant decrease	mesocosmos	Daam et al., 2009
Gerris latiabdominis	Admire	imidacloprid	49 µg/L (measured)	significant decrease	mesocosmos	Hayasaka et al., 2012a
Saldidae sp.	Admire	imidacloprid	49 µg/L (measured)	significant decrease	mesocosmos	Hayasaka et al., 2012a

Gerris latiabdominis	Prince	fipronil	<1 μg/L (measured)	no effect	mesocosmos	Hayasaka et al., 2012a
Saldidae sp.	Prince	fipronil	<1 μg/L (measured)	no effect	mesocosmos	Hayasaka et al., 2012a
Gerris latiabdominis	Admire	imidacloprid	49, 39 μg/L (measured)	no effect	mesocosmos	Hayasaka et al., 2012b
Notonecta triguttata	Admire	imidacloprid	49, 39 μg/L (measured)	no effect	mesocosmos	Hayasaka et al., 2012b
Gerris latiabdominis	Prince	fipronil	<1 μg/L (measured)	no effect	mesocosmos	Hayasaka et al., 2012b
Notonecta triguttata	Prince	fipronil	<1 μg/L (measured)	no effect	mesocosmos	Hayasaka et al., 2012b
Notonecta triguttata	Admire	imidacloprid	157.5 μg/L (measured)	significant decrease	mesocosmos	Kobashia et al., 2017
Notonecta triguttata	Starkle	dinotefuran	10.54 μg/L (measured)	no effect	mesocosmos	Kobashia et al., 2017
Notonecta sp.	Premier	imidacloprid	0.03 μg/L	no effect	mesocosmos	Sumon et al., 2018
Notonecta sp.	Premier	imidacloprid	0.3, 3.0 μg/L	significant decrease	mesocosmos	Sumon et al., 2018
Notonecta triguttata	Sainyoshi	pentoxazone	43.7 μg/L (measured)	marginally significant decrease	mesocosmos	Hashimoto et al., 2019
Microvelia douglasi	Sainyoshi	pentoxazone	43.7 μg/L (measured)	no effect	mesocosmos	Hashimoto et al., 2019
Gerris gracilicornis	Sainyoshi	pentoxazone	43.7 μg/L (measured)	no effect	mesocosmos	Hashimoto et al., 2019
Hydrometra procera	Sainyoshi	pentoxazone	43.7 μg/L (measured)	no effect	mesocosmos	Hashimoto et al., 2019
Gerromorpha sp.	Sainyoshi	pentoxazone	43.7 μg/L (measured)	no effect	mesocosmos	Hashimoto et al., 2019
Notonecta triguttata	Prince	fipronil	0.42 μg/L (measured)	no effect	mesocosmos	Hashimoto et al., 2019
Microvelia douglasi	Prince	fipronil	0.42 μg/L (measured)	no effect	mesocosmos	Hashimoto et al., 2019

Gerris gracilicornis	Prince	fipronil	0.42 µg/L (measured)	marginally significant decrease	mesocosmos	Hashimoto et al., 2019
Hydrometra procera	Prince	fipronil	0.42 µg/L (measured)	no effect	mesocosmos	Hashimoto et al., 2019
Gerromorpha sp.	Prince	fipronil	0.42 µg/L (measured)	no effect	mesocosmos	Hashimoto et al., 2019
Notonecta triguttata	Admire	imidacloprid	138.0 and 157.5 µg/L (measured)	significant increase	mesocosmos	Hayasaka et al., 2019
Notonecta triguttata	Starkle	dinotefuran	10.5 and 54.05 µg/L (measured)	no effect	mesocosmos	Hayasaka et al., 2019
Notonecta viridis		thiacloprid	0.46 µg/L (measured)	decrease	mesocosmos	Barmentlo et al., 2019
Gerris thoracicus		thiacloprid	0.46 µg/L (measured)	decrease	mesocosmos	Barmentlo et al., 2019
Cymatia coleoptrata		thiacloprid	0.46 µg/L (measured)	decrease	mesocosmos	Barmentlo et al., 2019
Sigara sp.		thiacloprid	0.46 µg/L (measured)	decrease	mesocosmos	Barmentlo et al., 2019
Notonecta viridis		thiacloprid+fertilizer	0.46 µg/L (measured) +higher N and P concentration	decrease	mesocosmos	Barmentlo et al., 2019
Gerris thoracicus		thiacloprid+fertilizer	0.46 µg/L (measured) +higher N and P concentration	decrease	mesocosmos	Barmentlo et al., 2019
Sigara lateralis		thiacloprid+fertilizer	0.46 µg/L (measured) +higher N and P concentration	increase	mesocosmos	Barmentlo et al., 2019
Cymatia coleoptrata		thiacloprid+fertilizer	0.46 µg/L (measured) +higher N and P concentration	increase	mesocosmos	Barmentlo et al., 2019
Gerris lacustris adults	Dantotsu	clothianidin	3.8, 5.7 and 17.1 µg/L (measured)	no effect	mesocosmos	Hashimoto et al., 2020
Gerris lacustris adults	Prince	fipronil	0.51, 0.13 and 0.53 µg/L (measured)	no effect	mesocosmos	Hashimoto et al., 2020

Gerris lacustris adults	Ferterra	chlorantraniliprole	4.2, 7.0 and 14.9 µg/L (measured)	no effect	mesocosmos	Hashimoto et al., 2020
Gerris lacustris nymph	Dantotsu	clothianidin	3.8, 5.7 and 17.1 µg/L (measured)	significant decrease	mesocosmos	Hashimoto et al., 2020
Gerris lacustris nymph	Prince	fipronil	0.51, 0.13 and 0.53 µg/L (measured)	no effect	mesocosmos	Hashimoto et al., 2020
Gerris lacustris nymph	Ferterra	chlorantraniliprole	4.2, 7.0 and 14.9 µg/L (measured)	no effect	mesocosmos	Hashimoto et al., 2020
Hydrometra procera adults	Dantotsu	clothianidin	3.8, 5.7 and 17.1 µg/L (measured)	no effect	mesocosmos	Hashimoto et al., 2020
Hydrometra procera adults	Prince	fipronil	0.51, 0.13 and 0.53 µg/L (measured)	no effect	mesocosmos	Hashimoto et al., 2020
Hydrometra procera adults	Ferterra	chlorantraniliprole	4.2, 7.0 and 14.9 µg/L (measured)	no effect	mesocosmos	Hashimoto et al., 2020
Hydrometra procera nimphs	Dantotsu	clothianidin	3.8, 5.7 and 17.1 µg/L (measured)	no effect	mesocosmos	Hashimoto et al., 2020
Hydrometra procera nimphs	Prince	fipronil	0.51, 0.13 and 0.53 µg/L (measured)	no effect	mesocosmos	Hashimoto et al., 2020
Hydrometra procera nimphs	Ferterra	chlorantraniliprole	4.2, 7.0 and 14.9 µg/L (measured)	no effect	mesocosmos	Hashimoto et al., 2020
Mesovelia thermalis adults	Dantotsu	clothianidin	3.8, 5.7 and 17.1 µg/L (measured)	no effect	mesocosmos	Hashimoto et al., 2020
Mesovelia thermalis adults	Prince	fipronil	0.51, 0.13 and 0.53 µg/L (measured)	no effect	mesocosmos	Hashimoto et al., 2020
Mesovelia thermalis adults	Ferterra	chlorantraniliprole	4.2, 7.0 and 14.9 µg/L (measured)	no effect	mesocosmos	Hashimoto et al., 2020
Microvelia douglasi adults	Dantotsu	clothianidin	3.8, 5.7 and 17.1 µg/L (measured)	no effect	mesocosmos	Hashimoto et al., 2020
Microvelia douglasi adults	Prince	fipronil	0.51, 0.13 and 0.53 µg/L (measured)	no effect	mesocosmos	Hashimoto et al., 2020
Microvelia douglasi adults	Ferterra	chlorantraniliprole	4.2, 7.0 and 14.9 µg/L (measured)	no effect	mesocosmos	Hashimoto et al., 2020
Microvelia douglasi nimphs	Dantotsu	clothianidin	3.8, 5.7 and 17.1 µg/L (measured)	no effect	mesocosmos	Hashimoto et al., 2020

Microvelia douglasi nimphs	Prince	fipronil	0.51, 0.13 and 0.53 μg/L (measured)	no effect	mesocosmos	Hashimoto et al., 2020
Microvelia douglasi nimphs	Ferterra	chlorantraniliprole	4.2, 7.0 and 14.9 μg/L (measured)	no effect	mesocosmos	Hashimoto et al., 2020
Saldidae spp. adults	Dantotsu	clothianidin	3.8, 5.7 and 17.1 μg/L (measured)	no effect	mesocosmos	Hashimoto et al., 2020
Saldidae spp. adults	Prince	fipronil	0.51, 0.13 and 0.53 μg/L (measured)	no effect	mesocosmos	Hashimoto et al., 2020
Saldidae spp. adults	Ferterra	chlorantraniliprole	4.2, 7.0 and 14.9 μg/L (measured)	no effect	mesocosmos	Hashimoto et al., 2020
Saldidae spp. nimphs	Dantotsu	clothianidin	3.8, 5.7 and 17.1 μg/L (measured)	no effect	mesocosmos	Hashimoto et al., 2020
Saldidae spp. nimphs	Prince	fipronil	0.51, 0.13 and 0.53 μg/L (measured)	no effect	mesocosmos	Hashimoto et al., 2020
Saldidae spp. nimphs	Ferterra	chlorantraniliprole	4.2, 7.0 and 14.9 μg/L (measured)	no effect	mesocosmos	Hashimoto et al., 2020
Sigara nigroventralis adults	Dantotsu	clothianidin	3.8, 5.7 and 17.1 μg/L (measured)	no effect	mesocosmos	Hashimoto et al., 2020
Sigara nigroventralis adults	Prince	fipronil	0.51, 0.13 and 0.53 μg/L (measured)	no effect	mesocosmos	Hashimoto et al., 2020
Sigara nigroventralis adults	Ferterra	chlorantraniliprole	4.2, 7.0 and 14.9 μg/L (measured)	no effect	mesocosmos	Hashimoto et al., 2020
Sigara substriata adults	Prince	fipronil	0.51, 0.13 and 0.53 μg/L (measured)	significant decrease	mesocosmos	Hashimoto et al., 2020
Sigara substriata adults	Ferterra	chlorantraniliprole	4.2, 7.0 and 14.9 μg/L (measured)	significant decrease	mesocosmos	Hashimoto et al., 2020
Sigara substriata nymphs	Ferterra	chlorantraniliprole	4.2, 7.0 and 14.9 μg/L (measured)	significant decrease	mesocosmos	Hashimoto et al., 2020
Corixa punctata		15 μm polystyrene MP	100 MPs/mL	no effect	mesocosmos	Al-Jaibachi et al., 2020
Notonecta sp.	Fenitrothion 50 EC	enitrothion	<25	significant decrease	mesocosmos	Mohammad Shadiqur Rahman et al., 2020

Ranatra linearis	Fenitrothion 50 EC	enitrothion	<25	significant decrease	mesocosmos	Mohammad Shadiqur Rahman et al., 2020
Gerris sp.	Fenitrothion 50 EC	enitrothion	<25	significant decrease	mesocosmos	Mohammad Shadiqur Rahman et al., 2020

Table S2b. Summary of the insecticide effects on water-bug species (semi-field and field studies), with LC50 determination.

species	pollutant		LC50 (µg/L)				author
	commercial name	active ingredient	24 h	48 h	96 h	168 h	
Sigara alternata	Thiodan 50 WP	endosulfan	13.0				Ernst et al. 1991
Sigara alternata	Thiodan 50 WP	endosulfan	75.0 (27.0-123.0)				Ernst et al. 1991
Sigara alternata	Thiodan 50 WP	endosulfan	269.0 (59.0-479.0)				Ernst et al. 1991
Sigara striata		lindane			3.9 (3.2-4.8)	2.2 (1.5-3.2)	Maund et al. 1992
Sigara substriata		pyridaphenthion	192 (172–218)	91 (81–104)			Takahashi et al. 2007
Anisops ogasawarensis		pyridaphenthion	460 (380–600)	180 (140–220)			Takahashi et al. 2007
Sigara substriata		pretilachlor		>4000			Takahashi et al. 2007
Anisops ogasawarensis		pretilachlor		>4000			Takahashi et al. 2007

Table S3. Effects of the microorganism derived pesticides.

Laboratory studies	Bti serotype	effect	remarks	author
Notonecta undulata				
mortality	ABG 6144	no	fed with intoxicated mosquito larvae	Aly and Mulla, 1987
length of the praeoviposition period (days)	ABG 6144	no	fed with intoxicated mosquito larvae	Aly and Mulla, 1987
egg number (No)	ABG 6144	no	fed with intoxicated mosquito larvae	Aly and Mulla, 1987
time till hatching (days)	ABG 6144	no	fed with intoxicated mosquito larvae	Aly and Mulla, 1987
consumption	ABG 6144	yes	fed with intoxicated mosquito larvae	Aly and Mulla, 1987
Notonecta sp.				
mortality	Teknar HP-D	no	fed with intoxicated mosquito larvae	Gunasekaran et al., 2004
consumption	Teknar HP-D	no	fed with intoxicated mosquito larvae	Gunasekaran et al., 2004
Diplonychus indicus				
mortality	Teknar HP-D	no	fed with intoxicated mosquito larvae	Gunasekaran et al., 2004
consumption	Teknar HP-D	no	fed with intoxicated mosquito larvae	Gunasekaran et al., 2004
Notonecta sp. nymph				
mortality	H-14	no	dose-response, from 1 to 7 days	Ser and Cetin, 2015
Buenoa tarsalis				
mortality	Bt-Horus SC	no		Gutiérrez et al., 2017
predatory abilities	Bt-Horus SC	no		Gutiérrez et al., 2017
Field studies				
Corisella spp.	H-14 deBarjac	no		Miura et al., 1980
Notonecta unifasciata	H-14 deBarjac	no		Miura et al., 1980
Buenoa scimitra	H-14 deBarjac	no		Miura et al., 1980
Notonecta indica	665 PM 50	yes		Purcell, 1981
Belastoma testaceum	666 PM 50	no		Purcell, 1981
Mesovelia amoena	667 PM 50	no		Purcell, 1981
M. mulsanti	668 PM 50	no		Purcell, 1981
Buenoa scimitra	SAN 402 WDC	no		Garcia et al., 1981
Notonecta kirbyi	Bactimos LRB 676	no		Garcia et al., 1981
Microvelia sp.	SAN 402 WDC	no		Garcia et al., 1981
Notonecta sp.	666 PM 52	no		Sebastien and Brust, 1981
Buenoa margaritacea	ABG 6511	no		Marina et al., 2014
Hydrometra wileyae	ABG 6512	no		Marina et al., 2014
Platyvelia brachialis	ABG 6513	no		Marina et al., 2014

Table S4. Effects of the plant originated insecticides. UCL = 95% upper confidence limit, LCL = 95% lower confidence limit. Remarks: the duration of the experiment.

species	plant name	acive substance	LC50 (µg/mL) (LCL–UCL)	remarks	author
Notonecta sp.	Noxfish	rotenone	3420.0 (2270.0-5150.0)	24 h	Chandler and Marking, 1982
Notonecta sp.	Noxfish	rotenone	1580.0 (727.0-3440.0)	96 h	Chandler and Marking, 1982
Diplonychus indicus	Atlantia monophylla		5650.0	24 h	Sivagnaname and Kalyanasundaram, 2004
Anisops bouvieri	Atlantia monophylla		140.0	24 h	Sivagnaname and Kalyanasundaram, 2004
Diplonychus rusticus	Sapindus emarginatus		9290.0	24 h	Koodalingam et al., 2009
D. rusticus	Sapindus emarginatus		8260.0	48 h	Koodalingam et al., 2009
Sphaerodema rusticum	Nimbecidine EC	azadirachtin	2.8 (2.5-3.0)	96 h	Shoba et al., 2011
Notonecta sp.	Euphorbia lactea extract		168.77	24 h	Samidurai and Mathew, 2014
Nepa cinerea	Euphorbia lactea extract		193.09	24 h	Samidurai and Mathew, 2014
A. bouvieri	Heracleum sprengelianum essential oil		1840.0	24 h	Govindarajan and Benelli, 2016a
D. indicus	Heracleum sprengelianum essential oil		2650.0	24 h	Govindarajan and Benelli, 2016a
A. bouvieri	Heracleum sprengelianum	lavandulyl acetate	206	24 h	Govindarajan and Benelli, 2016a
D. indicus	Heracleum sprengelianum	lavandulyl acetate	336.2	24 h	Govindarajan and Benelli, 2016a
A. bouvieri	Heracleum sprengelianum	bicyclogermacrene	414.0	24 h	Govindarajan and Benelli, 2016a
D. indicus	Heracleum sprengelianum	bicyclogermacrene	678.7	24 h	Govindarajan and Benelli, 2016a
A. bouvieri	Artemisia absinthium essential oil		3132.7	48 h	Govindarajan and Benelli, 2016b
A. bouvieri	Artemisia absinthium	(E)-β-farnesene	637.4	48 h	Govindarajan and Benelli, 2016b
A. bouvieri	Artemisia absinthium	(Z)-en-yn-dicycloether	1719.5	48 h	Govindarajan and Benelli, 2016b

A. bouvieri	Artemisia absinthium	(Z)-β-ocimene	2010.4	48 h	Govindarajan and Benelli, 2016b
A. bouvieri	green-synthesized silver nanoparticles using the Barleria cristata leaf extract		684.3 (615.3–746.8)	48 h	Govindarajan and Benelli, 2016c
D. indicus	green-synthesized silver nanoparticles using the Barleria cristata leaf extract		633.3 (562.5–696.1)	48 h	Govindarajan and Benelli, 2016c
A. bouvieri	Quisqualis indica-synthesized silver nanoparticles		653.1 (581.2–717.2)	48 h	Govindarajan et al., 2016a
D. indicus	Quisqualis indica-synthesized silver nanoparticles		861.0 (768.5–943.6)	48 h	Govindarajan et al., 2016a
A. bouvieri	Zornia diphylla aqueous leaf extract		4442.1 (3983.5–4856.1)	48 h	Govindarajan et al., 2016b
A. bouvieri	Zornia diphylla aqueous leaf extract green-fabricated silver nanoparticles		1100.7 (982.5–1207.0)	48 h	Govindarajan et al., 2016b
A. bouvieri	Ichnocarpus frutescens aqueous leaf extract		8412.4 (7436.6–9273.1)	48 h	Govindarajan et al., 2016c
D. indicus	Ichnocarpus frutescens aqueous leaf extract		10738.0 (9566.4–11783.2)	48 h	Govindarajan et al., 2016c
A. bouvieri	silver nanoparticles fabricated using the Ichnocarpus frutescens leaf extract		636.6 (567.1–698.6)	48 h	Govindarajan et al., 2016c
D. indicus	silver nanoparticles fabricated using the Ichnocarpus frutescens leaf extract		873.3 (775.3–960.3)	48 h	Govindarajan et al., 2016c
A. bouvieri	Pinus kesiya essential oil		4135.0	48 h	Govindarajan et al., 2016d
D. indicus	Pinus kesiya essential oil		4545.0	48 h	Govindarajan et al., 2016d
A. bouvieri	Origanum scabrum essential oil		4162.1	24 h	Govindarajan et al., 2016e
D. indicus	Origanum scabrum essential oil		5454.1	24 h	Govindarajan et al., 2016e
D. indicus	Clerodendrum chinense aqueous leaf extract		3110.8 (2764.8–3418.2)	48 h	Govindarajan et al., 2016f
A. bouvieri	Clerodendrum chinense aqueous leaf extract		358.5 (3909.5–4763.6)	48 h	Govindarajan et al., 2016f
D. indicus	green-synthesized silver nanoparticles		647.0 (579.0–708.2)	48 h	Govindarajan et al., 2016f
A. bouvieri	green-synthesized silver nanoparticles		89.5 (792.2–976.3)	48 h	Govindarajan et al., 2016f
A. bouvieri	Carissa spinarum aqueous leaf extract		4475.2 (4002.5–4900.3)	48 h	Govindarajan et al., 2016g
D. indicus	Carissa spinarum aqueous leaf extract		4145.6 (3682.5–4556.6)	48 h	Govindarajan et al., 2016g

A. bouvieri	Ichnocarpus frutescens aqueous leaf extract	8412.4 (7436.6–9273.1)	48 h	Govindarajan et al., 2016h
D. indicus	Ichnocarpus frutescens aqueous leaf extract	10738.0 (9566.4-11783.2)	48 h	Govindarajan et al., 2016h
A. bouvieri	silver nanoparticles fabricated using the Ichnocarpus frutescens leaf extract	636.6 (567.1–698.6)	48 h	Govindarajan et al., 2016h
D. indicus	silver nanoparticles fabricated using the Ichnocarpus frutescens leaf extract	873.3 (775.3–960.3)	48 h	Govindarajan et al., 2016h
D. indicus	Nicandra physalodes aqueous leaf extract	19,076.59 (17,014.79–20,918.34)	48 h	Govindarajan et al., 2016i
D. indicus	green-synthesized silver nanoparticles	1032.81 (916.83–1135.66)	48 h	Govindarajan et al., 2016i
A. bouvieri	aqueous leaf extract Hedyotis puberula	12365.0 (10955.1-13612.5)	24 h	Azarudeen et al., 2016
D. indicus	aqueous leaf extract Hedyotis puberula	18747.8 (16647.8-20610.2)	24 h	Azarudeen et al., 2016
A. bouvieri	green-synthesized silver nanoparticles using the leaf extr Hedyotis puberula	1048.1 (934.1-1149.7)	24 h	Azarudeen et al., 2016
D. indicus	green-synthesized silver nanoparticles using the leaf extr Hedyotis puberula	1658.8 (1476.5-1821.2)	24 h	Azarudeen et al., 2016
A. bouvieri	Carissa carandas aqueous leaf extract	9084.5 (8204.0–9889.7)	48 h	Govindarajan and Benelli, 2017
D. indicus	Carissa carandas aqueous leaf extract	10485.1 (9556.9–1136.4)	48 h	Govindarajan and Benelli, 2017
A. bouvieri	green-synthesized silver nanoparticles using the Carissa carandas leaf extract	1097.9 (692.7-1386.6)	48 h	Govindarajan and Benelli, 2017
D. indicus	green-synthesized silver nanoparticles using the Carissa carandas leaf extract	1293.6 (1179.4-1401.4)	48 h	Govindarajan and Benelli, 2017
A. bouvieri	Rubus ellipticus aqueous leaf extract	13,301.3 (11,911.1–14,551.9)	48 h	AlQahtani et al., 2017
D. indicus	Rubus ellipticus aqueous leaf extract	18,367.7 (16,658.2–19,938.7)	48 h	AlQahtani et al., 2017
A. bouvieri	Rubus ellipticus aqueous leaf extract synthesized silver nanoparticles	896.7 (807.8–977.4)	48 h	AlQahtani et al., 2017

D. indicus	Rubus ellipticus aqueous leaf extract synthesized silver nanoparticles	1348.8 (1215.9–1489.7)	48 h	AlQahtani et al., 2017
A. bouvieri	Aganosma cymosa aqueous leaf extract	12980.6 (11648.6–14181.2)	48 h	Benelli and Govindarajan, 2017
D. indicus	Aganosma cymosa aqueous leaf extract	15692.4 (14109.9–17124.6)	48 h	Benelli and Govindarajan, 2017
A. bouvieri	green-synthesized silver nanoparticles using the Aganosma cymosa leaf extract	673.4 (605.4–734.9)	48 h	Benelli and Govindarajan, 2017
D. indicus	green-synthesized silver nanoparticles using the Aganosma cymosa leaf extract	907.7 (821.6–986.5)	48 h	Benelli and Govindarajan, 2017
A. bouvieri	Hugonia mystax aqueous leaf extract against	12251.7 (10819.3–13512.4)	48 h	Govindarajan et al., 2017a
D. indicus	Hugonia mystax aqueous leaf extract against	14756.4 (13094.3–16230.7)	48 h	Govindarajan et al., 2017a
A. bouvieri	synthesized AgNPs using the Hugonia mystax leaf extract	829.6 (734.0–914.1)	48 h	Govindarajan et al., 2017a
D. indicus	synthesized AgNPs using the Hugonia mystax leaf extract	1075.2 (956.8–1180.6)	48 h	Govindarajan et al., 2017a
D. indicus	Adiantum raddianum aqueous leaf extract	6329.5 (5641.2–6943.4)	48 h	Govindarajan et al., 2017b
D. indicus	silver nanocrystals synthesized using the Adiantum raddianum leaf extract	517.9 (460.6–568.8)	48 h	Govindarajan et al., 2017b
A. bouvieri	M. emarginata aqueous leaf extract	8317.5 (7392.9–9138.7)	48 h	Azarudeen et al., 2017a
D. indicus	M. emarginata aqueous leaf extract	13,081.1 (11,599.6–14,395.2)	48 h	Azarudeen et al., 2017a
A. bouvieri	M. emarginata-synthesized silver nanoparticles	415.6 (369.7–456.5)	48 h	Azarudeen et al., 2017a
D. indicus	M. emarginata-synthesized silver nanoparticles	633.5 (559.3–698.9)	48 h	Azarudeen et al., 2017a
A. bouvieri	Naregamia alata aqueous leaf extract	10,409.3 (9246.2–11,441.9)	48 h	Azarudeen et al., 2017b
D. indicus	Naregamia alata aqueous leaf extract	14,644.3 (13,046.0–16,068.8)	48 h	Azarudeen et al., 2017b
A. bouvieri	silver nanoparticles green-synthesized using the Naregamia alata leaf extract	629.3 (561.5–690.4)	48 h	Azarudeen et al., 2017b
D. indicus	silver nanoparticles green-synthesized using the Naregamia alata leaf extract	1058.3 (948.1–1157.5)	48 h	Azarudeen et al., 2017b

A. bouvieri	silver nanoparticles fabricated using the Ichnocarpus frutescens leaf extract		831.8 (741.7–912.2)	48 h	Aarthi Chinnadurai et al., 2018
D. indicus	silver nanoparticles fabricated using the Ichnocarpus frutescens leaf extract		1286.9 (1148.6–1410.5)	48 h	Aarthi Chinnadurai et al., 2018
A. bouvieri	Zingiber cernuum essential oil		3119.3	48 h	Rajeswary et al., 2018
D. indicus	Zingiber cernuum essential oil		5273.8	48 h	Rajeswary et al., 2018
A. bouvieri	Amomum subulatum essential oil		3123.1	24 h	Govindarajan et al., 2018a
D. indicus	Amomum subulatum essential oil		4161.6	24 h	Govindarajan et al., 2018a
A. bouvieri	Eugenia uniflora essential oil		2025.6	48 h	Govindarajan et al., 2018b
D. indicus	Eugenia uniflora essential oil		3191.0	48 h	Govindarajan et al., 2018b
A. bouvieri	Eugenia uniflora	curzerene	303.8	48 h	Govindarajan et al., 2018b
D. indicus	Eugenia uniflora	curzerene	512.8	48 h	Govindarajan et al., 2018b
A. bouvieri	Eugenia uniflora	trans-β-elemenone	414.0	48 h	Govindarajan et al., 2018b
D. indicus	Eugenia uniflora	trans-β-elemenone	612.9	48 h	Govindarajan et al., 2018b
A. bouvieri	Eugenia uniflora	γ-elemene	829.1	48 h	Govindarajan et al., 2018b
D. indicus	Eugenia uniflora	γ-elemene	1031.4	24 h	Govindarajan et al., 2018b
A. bouvieri	Acacia caesia aqueous leaf extract		11,063.3 (9961.1–12,062.3)	48 h	Benelli et al., 2018a
D. indicus	Acacia caesia aqueous leaf extract		9012.2 (8164.3–9789.2)	48 h	Benelli et al., 2018a
A. bouvieri	Ag nanoparticles synthesized using Aglaia elaeagnoidea leaves		1247.7 (1110.3–1370.0)	48 h	Benelli et al., 2018b
D. indicus	Ag nanoparticles synthesized using Aglaia elaeagnoidea leaves		1895.0 (1693.0–2075.7)	48 h	Benelli et al., 2018b
A. bouvieri	Aglaia elaeagnoidea aqueous leaf extract		14,654.7 (13,051.6–16,083.4)	48 h	Benelli et al., 2018b
D. indicus	Aglaia elaeagnoidea aqueous leaf extract		20,915.8 (18,669.9–22,923.5)	48 h	Benelli et al., 2018b
A. bouvieri	Ag nanoparticles fabricated using the extract of Aglaia elaeagnoidea leaves		1247.7 (1110.3–1370.0)	48 h	Benelli et al., 2018b
D. indicus	Ag nanoparticles fabricated using the extract of Aglaia elaeagnoidea leaves		1895.0 (1693.0–2075.7)	48 h	Benelli et al., 2018b
A. bouvieri	Syzygium lanceolatum essential oil		4148.3	24 h	Benelli et al., 2018c
D. indicus	Syzygium lanceolatum essential oil		6189.3	24 h	Benelli et al., 2018c

A. bouvieri	Boswellia ovalifoliolata essential oil		4186.9	24 h	Benelli et al., 2018d
D. indicus	Boswellia ovalifoliolata essential oil		6210.3	24 h	Benelli et al., 2018d
A. bouvieri	Citrus limetta major phyto-compound		2146.8 (1877.3-2364.3)	48 h	Mathalaimuthu Baranitharana et al., 2020
D. indicus	Citrus limetta major phyto-compound		1819.6 (1526.8-1941.3)	48 h	Mathalaimuthu Baranitharana et al., 2020
A. bouvieri	Citrus limetta leaf methanolic extract		4133.6 (3782.2-4425.8)	48 h	Mathalaimuthu Baranitharana et al., 2020
D. indicus	Citrus limetta leaf methanolic extract		3664.8 (2942.9-3871.9)	48 h	Mathalaimuthu Baranitharana et al., 2020
A. bouvieri	Alstonia venenata aqueous leaf extract		6233.3 (5556.1–6837.0)	48 h	Esan et al., 2020
D. indicus	Alstonia venenata aqueous leaf extract		10,519.3 (9360.8–11,550.9)	48 h	Esan et al., 2020
A. bouvieri	Alstonia venenata derived AgNPs		734.7 (655.3–805.7)	48 h	Esan et al., 2020
D. indicus	Alstonia venenata derived AgNPs		1057.9 (938.3–1163.8)	48 h	Esan et al., 2020
D. rusticus	Conyza canadensis		135.7	24 h	Tran Minh Hoi et al., 2020
D. rusticus	Conyza sumatrensis		111.0	48 h	Tran Minh Hoi et al., 2020
D. rusticus	Conyza canadensis		124.0	24 h	Tran Minh Hoi et al., 2020
D. rusticus	Conyza sumatrensis		107.8	48 h	Tran Minh Hoi et al., 2020
A. bouvieri	Kaempferia galanga rhizome essential oil	Essential oil	2173.5 (1977.5-2358.9)	48 h	AlSalhi et al., 2020
A. bouvieri	Kaempferia galanga rhizome essential oil	Ethyl pmethoxy methoxycinnamate	715.4 (659.7-778.9)	48 h	AlSalhi et al., 2020
A. bouvieri	Kaempferia galanga rhizome essential oil	trans-Ethylcinnamate	1779.6 (1627.4-1930.3)	48 h	AlSalhi et al., 2020
A. bouvieri	Kaempferia galanga rhizome essential oil	trans-Cinnamaldehyde	1820.3 (1680.0-1960.8)	48 h	AlSalhi et al., 2020
A. bouvieri	Alstonia venenata aqueous leaf extract		6233.3 (5556.1–6837.0)	48 h	Esan et al., 2021
D. indicus	Alstonia venenata aqueous leaf extract		10,519.3 (9360.8–11,550.9)	48 h	Esan et al., 2021
A. bouvieri	Alstonia venenata derived AgNPs		734.7 (655.3–805.7)	48 h	Esan et al., 2021
D. indicus	Alstonia venenata derived AgNPs		1057.9 (938.3–1163.8)	48 h	Esan et al., 2021

Table S5a.

Results of laboratory dose-response studies with Cd, and Cd concentrations in animals collected from the field. Remarks: the duration of the experiment and the cadmium compound used. (a): wet season, (b): dry season, (1): control area, (2): close to an iron and steel factory. *: average and standard deviation calculated based on the original data.

Laboratory data	LC50 (mg/L)	Remarks	Author
Ranatra elongata	0.438	48 h, CdCl2	Shukla et al., 1983
Ranatra elongata	0.288	96 h, CdCl2	Shukla et al., 1983
Corixa punctata	>56	48 h, Cd(No3)2	Sloof, 1983
Anisops sardeus (V. nymph)(a)	0.9	96 h, CdCl2	Chanu et al., 2017
Anisops sardeus (female)(a)	0.59	96 h, CdCl2	Chanu et al., 2017
Anisops sardeus (male)(a)	0.51	96 h, CdCl2	Chanu et al., 2017
Anisops sardeus (female)(b)	26.7	96 h, CdCl2	Chanu et al., 2017
Anisops sardeus (male)(b)	20.2	96 h, CdCl2	Chanu et al., 2017
Field data	Cd µg/g dry weight (±SD)		
Halobates sobrinus	151.2 (± 54.6)		Cheng et al., 1976
Halobates sericeus	65.3 (± 13.6)		Cheng et al., 1976
Rheumatobates	< 5		Cheng et al., 1976
Halobates micans	1.7-122		Schulz-Baldes and Cheng, 1980
Sigara sp.	0.034 (± 0.03)		Barak and Mason, 1989
Notonectidae	1.2 (± 0.1 SE)		Scheuhammer et al. 1997
Gerridae	1.0 (± 0.1 SE)		Scheuhammer et al. 1997
Gerris lateralis (1)	5.02 (± 0.9)*		Nummelin et al., 1998
Gerris lateralis (2)	1.83 (± 0.2)*		Nummelin et al., 1998
Gerris odontogaster (1)	10.4 (± 2.0)*		Nummelin et al., 1998
Gerris odontogaster (2)	1.9 (± 0.3)*		Nummelin et al., 1998
Gerris thoracicus (1)	4.3 (± 2.5)*		Nummelin et al., 1998
Gerris thoracicus (2)	1.05 (± 0.2)*		Nummelin et al., 1998
Gerris argentatus (1)	9.0 (± 1.6)*		Nummelin et al., 1998
Anisops sardeus	1.5 (± 0.1)		Ahmed and El-Shenawy 2001
Anisops sardeus	2.7 (± 0.2)		Ahmed and El-Shenawy 2001

Table S5b.

Hg concentrations in animals collected from the field. THg: total mercury concentration, MeHg: methylmercury concentration.

Species	THg µg/g dry weigh (±SD)t	MeHg µg/g dry weight (±SD)	THg µg/g wet weight	Remarks	Author
Ranatra sp.			2.82		Vermeer et al., 1973
Corixidae			5.2		Vermeer et al., 1973
Sigara	0.034 ±0.003				Barak and Mason, 1989
Gerris sp. (Lake group1)	0.180 ±0.1	0.18 ±0.14			Tremblay et al., 1996
Gerris sp. (Lake group2)	0.20 ±0.15	0.18 ±0.12			Tremblay et al., 1996
Sigara sp. (Lake group1)	0.28 ±0.18	0.15 ±0.13			Tremblay et al., 1996
Sigara sp. (Lake group2)	0.27 ±0.13	0.17 ±0.12			Tremblay et al., 1996
Lethocerus americanus (L979,1992)		0.499 ±0.03			Hall et al., 1998
Lethocerus americanus (L979,1993)		0.108		one individual	Hall et al., 1998
Lethocerus americanus (L632,1992)		0.429 ±0.04			Hall et al., 1998
Lethocerus americanus (L632,1993)		0.311 ±0.07			Hall et al., 1998
Lethocerus americanus (L632,1994)		0.094 ±0.005			Hall et al., 1998
Corixidae (L979, 1993)		0.318 ±0.03			Hall et al., 1998
Corixidae (1994a)		0.285 ±0.003		drawdown sample	Hall et al., 1998
Corixidae (1994b)		0.157 ±0.017		reflood sample	Hall et al., 1998
Corixidae (L632, 1992)		0.121		1 individual	Hall et al., 1998
Corixidae (L632, 1993)		0.156 ±0.022			Hall et al., 1998
Corixidae (L632, 1994)		0.124 ±0.022			Hall et al., 1998
Gerris sp. (L979, 1993a)		0.109		preflood sample, 1 individual	Hall et al., 1998
Gerris sp. (L979, 1993b)		0.405 ±0.118		postflood sample	Hall et al., 1998
Gerris sp. (L979, 1994)		0.181 ±0.104			Hall et al., 1998
Gerris sp. (L632, 1993)		0.142 ±0.047			Hall et al., 1998
Gerris sp. (L240, 1993)		0.119 ±0.007			Hall et al., 1998
Gerris sp. (L240, 1994)		0.151 ±0.012			Hall et al., 1998
Notonecta sp. (L979, 1993a)		0.159 ±0.134		preflood sample	Hall et al., 1998

Notonecta sp. (L979, 1993b)		0.260 ±0.028	postflood sample	Hall et al., 1998
Notonecta sp. (L979, 1994a)		0.331	drawdown sample	Hall et al., 1998
Notonecta sp. (L979, 1994b)		0.442 ±0.060	reflood sample	Hall et al., 1998
Notonecta sp. (L362, 1992)		0.430	1 individual	Hall et al., 1998
Notonecta sp. (L362, 1993)		0.182 ±0.025		Hall et al., 1998
Notonecta sp. (L362, 1994)		0.235 ±0.050		Hall et al., 1998
Ranatra sp. (L362, 1992)		0.334 ±0.001		Hall et al., 1998
Ranatra sp. (L362, 1993)		0.233	1 individual	Hall et al., 1998
Ranatra sp. (L362, 1994)		0.366 ±0.137		Hall et al., 1998
Belostoma sp. (F1, 1995)		0.003		Cleckner et al., 1998
Belostoma sp. (F1, 1996)		0.001		Cleckner et al., 1998
Belostoma sp. (U3, 1996)		0.061		Cleckner et al., 1998
Gerridae (GCU)	7.28 ±0.43			Jardine et al., 2005
Gerridae (18M)	0.57 ±0.2			Jardine et al., 2005
Gerridae (18B)	0.50 ±0.15			Jardine et al., 2005
Gerridae (18A)	0.32 ±0.1			Jardine et al., 2005
Gerridae (DEB)	0.47 ±0.08			Jardine et al., 2005
Gerridae (NWA)	0.26 ±0.1			Jardine et al., 2005
Gerridae (HAB, May)	0.80 ±0.33			Jardine et al., 2005
Gerridae (HAB, July)	1.4 ±0.2			Jardine et al., 2005
Gerridae (MCB, May)	0.53 ±0.15			Jardine et al., 2005
Gerridae (MCB, July)	0.61 ±0.07			Jardine et al., 2005
Gerridae (PLB, May)	0.29 ±0.04			Jardine et al., 2005
Gerridae (PLB, July)	0.35 ±0.07			Jardine et al., 2005
Notonecta spp.		0.175 ±0.012		Allen et al., 2005
Corixidae (AT)	0.05 ±0.05			Tavares et al., 2008
Corixidae (VP-a)	0.05 ±0.02		a,b,c: temporal replicates	Tavares et al., 2008
Corixidae (VP-b)	0.13 ±0.05		a,b,c: temporal replicates	Tavares et al., 2008
Corixidae (VP-c)	0.10 ±0.04		a,b,c: temporal replicates	Tavares et al., 2008
Corixidae (VS)	0.09 ±0.05			Tavares et al., 2008

Corixidae (BP)	0.03 ±0.001			Tavares et al., 2008
Corixidae (PS)	0.08 ±0.02			Tavares et al., 2008
Corixidae (VAI-a)	0.06 ±0.01		a,b,c: temporal replicates	Tavares et al., 2008
Corixidae (VAI-b)	0.04 ±0.01		a,b,c: temporal replicates	Tavares et al., 2008
Corixidae (VAI-c)	0.04 ±0.02		a,b,c: temporal replicates	Tavares et al., 2008
Corixidae (GA)	0.04 ±0.01			Tavares et al., 2008
Corixidae (CP)	0.02 ±0.02			Tavares et al., 2008
Corixidae (CM)	0.12 ±0.04			Tavares et al., 2008
Corixidae (Vaia, 2001, C1)	0.05 ±0.01		C1,C2,C3: spetial replicates	Tavares et al., 2008
Corixidae (Vaia, 2001, C2)	0.05 ±0.02		C1,C2,C3: spetial replicates	Tavares et al., 2008
Corixidae (Vaia, 2001, C3)	0.03 ±0.001		C1,C2,C3: spetial replicates	Tavares et al., 2008
Corixidae (Vau, 2001)	0.15 ±0.02			Tavares et al., 2008
Corixidae (Vaia, 2002, C2)	0.04 ±0.02			Tavares et al., 2008
Corixidae (Vau, 2002)	0.11 ±0.01			Tavares et al., 2008
Corixidae (P.Sado, 2002)	0.08 ±0.01			Tavares et al., 2008
Belostoma sp.	0.143 ±0.023	0.121 ±0.025	8 sampling time in two years, 4 sampling site	Cremona et al., 2008
Callicorixa sp.	0.113 ±0.008	0.099 ±0.01		Cremona et al., 2008
Gerris sp.	0.176	-	2 individuals	Cremona et al., 2008
Ranatra sp.	0.406	0.378	1 individual	Cremona et al., 2008
Notonecta sp.	0.236 ±0.02	0.242 ±0.016		Cremona et al., 2008
Neoplea sp.	0.162 ±0.012	0.150 ±0.012		Cremona et al., 2008
Mesovelidae	0.159 ±0.032	0.163 ±0.031		Cremona et al., 2008
Aquarius remigis (SW)	0.26 ±0.05		with Metrobates hesperius in some places	Jardine et al., 2009
Aquarius remigis (LSJ)	0.22 ±0.06		with Metrobates hesperius in some places	Jardine et al., 2009
Aquarius remigis (IFB)	0.20 ±0.03		with Metrobates hesperius in some places	Jardine et al., 2009
Aquarius remigis (USJ)	0.15 ±0.04		with Metrobates hesperius in some places	Jardine et al., 2009
Aquarius remigis (MIR)	0.14 ±0.01		with Metrobates hesperius in some places	Jardine et al., 2009

Aquarius remigis (SE)	0.14 ±0.02		with Metrobates hesperius in some places	Jardine et al., 2009
Aquarius remigis (REST)	0.13 ±0.02		with Metrobates hesperius in some places	Jardine et al., 2009
Aquarius remigis (CHA)	0.13 ±0.03		with Metrobates hesperius in some places	Jardine et al., 2009
Aquarius remigis (Grand Lake)	0.35 ±0.06		with Metrobates hesperius in some places	Jardine et al., 2009
Aquarius remigis (Belledune)	0.15 ±0.02		with Metrobates hesperius in some places	Jardine et al., 2009
Corixidae	0.111-0.248			Blackwell and Drenner, 2009
Notonectidae	0.260-0.959			Blackwell and Drenner, 2009
Micronecta scholtzi (B)	3.6	0.72	high Hg concentration in sediment	Agra et al., 2010
Micronecta scholtzi (C)	12.6	2.5	high Hg concentration in sediment	Agra et al., 2010
Micronecta scholtzi (D)	12.0	2.4	high Hg concentration in sediment	Agra et al., 2010
Micronecta scholtzi (DJ)	11.7	2.3	high Hg concentration in sediment	Agra et al., 2010
Micronecta scholtzi (DM)	1.6	0.32	low Hg concentration in sediment	Agra et al., 2010
Micronecta scholtzi (E)	0.2	0.04	low Hg concentration in sediment	Agra et al., 2010
Corisella (WR-I)	0.58 ±0.19			Ackerman et al., 2010
Corisella (WR-C)	0.87 ±0.24			Ackerman et al., 2010
Corisella (WR-O)	1.02 ±0.14			Ackerman et al., 2010
Corisella (WdR-I)	0.82 ±0.10			Ackerman et al., 2010
Corisella (WdR-C)	0.66 ±0.11			Ackerman et al., 2010
Corisella (WdR-O)	0.84 ±0.04			Ackerman et al., 2010
Corisella (PW-I)	0.82 ±0.09			Ackerman et al., 2010
Corisella (PW-C)	0.84 ±0.12			Ackerman et al., 2010
Corisella (PW-O)	0.83 ±0.25			Ackerman et al., 2010
Corisella (FF-I)	0.88 ±0.15			Ackerman et al., 2010
Corisella (FF-C)	1.08 ±0.18			Ackerman et al., 2010
Corisella (FF-O)	1.26 ±0.15			Ackerman et al., 2010
Notonecta (WR-I)	0.86 ±0.09			Ackerman et al., 2010
Notonecta (WR-C)	0.94 ±0.23			Ackerman et al., 2010

Notonecta (WR-O)	1.49 ±0.27		Ackerman et al., 2010
Notonecta (WdR-I)	0.88 ±0.28		Ackerman et al., 2010
Notonecta (WdR-C)	0.73 ±0.09		Ackerman et al., 2010
Notonecta (WdR-O)	0.84 ±0.19		Ackerman et al., 2010
Notonecta (PW-I)	1.72 ±0.11		Ackerman et al., 2010
Notonecta (PW-C)	2.55 ±0.02		Ackerman et al., 2010
Notonecta (PW-O)	1.88 ±0.30		Ackerman et al., 2010
Notonecta (FF-I)	1.15 ±0.09		Ackerman et al., 2010
Notonecta (FF-C)	1.12 ±0.15		Ackerman et al., 2010
Notonecta (FF-O)	1.17 ±0.05		Ackerman et al., 2010
Corisella (WR-Fu)	0.88 ±0.18		Ackerman et al., 2010
Corisella (WR-Ph)	0.72 ±0.12		Ackerman et al., 2010
Corisella (WdR-Fu)	0.73 ±0.08		Ackerman et al., 2010
Corisella (WdR-Ph)	0.76 ±0.07		Ackerman et al., 2010
Corisella (PW-Fu)	0.78 ±0.10		Ackerman et al., 2010
Corisella (PW-Ph)	0.81 ±0.15		Ackerman et al., 2010
Corisella (FF-Fu)	1.22 ±0.14		Ackerman et al., 2010
Corisella (FF-Ph)	0.88 ±0.08		Ackerman et al., 2010
Notonecta (WR-Fu)	0.88 ±0.24		Ackerman et al., 2010
Notonecta (WR-Ph)	1.25 ±0.12		Ackerman et al., 2010
Notonecta (WdR-Fu)	0.52 ±0.04		Ackerman et al., 2010
Notonecta (WdR-Ph)	1.05 ±0.14		Ackerman et al., 2010
Notonecta (PW-Fu)	1.42 ±0.50		Ackerman et al., 2010
Notonecta (PW-Ph)	2.15 ±0.15		Ackerman et al., 2010
Notonecta (FF-Fu)	1.12 ±0.07		Ackerman et al., 2010
Notonecta (FF-Ph)	1.10 ±0.10		Ackerman et al., 2010
Corisella (WR)		0.650 ±0.076	Ackerman et al., 2010
Corisella (WdR)		0.643 ±0.055	Ackerman et al., 2010
Corisella (PW)		0.894 ±0.138	Ackerman et al., 2010
Corisella (FF)		1.001 ±0.096	Ackerman et al., 2010

Belostoma sp.	0.346 ±0.071	0.294 ±0.068		Chumchal et al., 2011
Notonectidae (UR1)		0.054	site identification code in parenthese	Williams et al., 2011
Belastomatidae (UR1)		0.048 ±0.002		Williams et al., 2011
Naucoridae (UR1)		0.014		Williams et al., 2011
Naucoridae (UR2)		0.056		Williams et al., 2011
Nepidae (DM2)		0.251		Williams et al., 2011
Notonectidae (UI1)		0.24		Williams et al., 2011
Naucoridae (UI1)		0.012		Williams et al., 2011
Corixidae (UI1)		0.08 ±0.002		Williams et al., 2011
Hesperocorixa spp.	0.170 ±0.02			Henderson et al., 2012
Ranatra spp.	0.191 ±0.005			Henderson et al., 2012
Belostoma spp.	0.173 ± 0.005			Henderson et al., 2012
Buenoa spp.	0.491 ±0.10			Henderson et al., 2012
Notonecta spp.	0.816 ±0.17			Henderson et al., 2012
Sigara semistriata and S. fossarum		0.095-0.121	composite samples	Lindholm et al., 2014
Gerris remigis	0.134-0.211	0.113-0.191		Tsui et al., 2014

Table S6. Occurrence of species in standing waters differing in trophic level. Numbers refer to the papers: 1.: Macan (1954), 2.: Savage (1994), 3.: Verberk (2005), 4.: Silling és Urbanic (2016), 5.: Kurzatkowska (2003), 6.: Jansson (1977b), 7.: Gerend (2006), 8.: Carbonell (2011), 9.: Vásárhelyi, Bakonyi (2012)

Table S6a	oligotroph	mesotroph	eutroph
Aphelocheirus aestivalis	4		
Corixa punctata	3	3	1
Callicorixa concinna			2
Sigara nigrolineata			1
Sigara lateralis			1
S. falleni			2
Hesperocorixa linnei			3
H. sahlbergi			1
I. cimicoides		4	
N. cinerea	4		
R. linearis			3
P. minutissima			4, 5
Hydrometra glacilenta		3	3
Hebrus pusillus			3
Microvelia reticulata		3	3, 5
M. umbricola		3	3
Mesovelia furcata			4
Gerris argentatus			3
G. lacustris	3		
G. odontogaster	3	3	
Table S6b			
Micronecta griseola	9	5	6
M. minutissima		6	5
M. poweri	5, 6		
M. scholtzi			7, 8, 9
M. pusilla	9		

Table S7

Corixidae species occurring in saline water of different concentrations. Numbers refer to the papers: 1.: Sowa et al. (2018), 2.: Carbonell et al. (2012), 3.: Golovatyuk and Shitikov (2016), 4.: Velasco et al. (2006), 5.: Barahona et al. (2005), 6.: Savage (1974), 7.: Kelts (1979), 8.: Knowles and Williams (1973), 9.: Gunter and Christmas (1959), 10.: Tones and Hammer (1975), 11.: Fouzi et al. (2020), 12.: Carbonell et al. (2015), 13.: Jang and Tullis (1980), 14.: Sanguinetti (1980), 15.: Gallardo-Mayenco (1994), 16.: Zinchenko and Golovatyuk (2013), 17.: Boda et al. (2019), 18.: Lancaster and Scudder (1987). Classification of water bodies salt concentration after Woynarovich et al. 2020.

Species	Diluted salt waters (1 000-5 000 mg/L)	Moderate salt waters (5 000-18 000 mg/L)	Concentrated salt waters (18 000-30 000 mg/L)	Very concentrated salt waters (30 000-40 000 mg/L)	Hypersaline brine waters (> 40 000 mg/L)
Agraptocorixa eurynome	8				
Agraptocorixa hirtifrons		8			
Callicorixa gebleri		3			
Caenocorixa bifida		18			
Caenocorixa expleta		18			
Paracorixa concinna		6, 16, 17			
Sigara australis		8			
Sigara assimilis				1, 3, 16	
Sigara lateralis	12	3, 6, 16, 17			
Sigara falleni	6				
Sigara nigrolineata				16	
Sigara scripta		2	12		
Sigara selecta		15	2, 6	5	4, 12
Sigara stagnalis		6			
Trichocorixa reticulata					13, 14
Trichocorixa verticalis				7, 9, 12	10, 11

References in supplementary material only

Aarthi, C., Govindarajan, M., Rajaraman, P., Alharbi, N. S., Kadaikunnan, S., Khaled, J. M., Mothana, R. A., et al. (2018). Eco-friendly and cost-effective Ag nanocrystals fabricated using the leaf extract of Habenaria plantaginea: toxicity on six mosquito vectors and four non-target species. *Environmental Science and Pollution Research*, *25*(11), 10317–10327. https://doi.org/10.1007/s11356-017-9203-2

Ahmed, A., Ahmed, S., Madsen, H. & Appleton, C. (2003). Extracts of leaves and seeds of the Neem tree, Azadirachta indica, as environment-oriented molluscicides for combating schistosomiasis. *Proceedings of Workshop on African Freshwater Malacology*, 9–12.

Ahmed, R. & El-Shenawy, N. (2001). Anisops sardeus Herrich-Schaeffer (Heteroptera: Notonectidae) as a bioindicator of heavy metals in wastewater treatment plant at Ismailiya, Egypt. *Egyptian Journal of Aquatic Biology and Fisheries*, *5*(2), 129–146. https://doi.org/10.21608/ejabf.2001.1683

Alexander, T., TC, A., WB, K. & AL, A. (1982). Effect of notonectids on mosquito larvae and preliminary observations of insecticide toxicity. *Arkansas Farm Research*, *31*(3), 5.

Ali, H., Abed, I., Augul, R. & Fadhil, H. (2020). Insecticidal activity of Eucalyptus sp. volatile oil against backswimmer insect Anisops sardea. *The Iraqi Journal of Agricultural Science*, *51*(1), 470–482. https://doi.org/10.36103/ijas.v51i1.946

Aljaibachi, R., Laird, W. B., Stevens, F. & Callaghan, A. (2020). Impacts of polystyrene microplastics on Daphnia magna: A laboratory and a mesocosm study. *Science of The Total Environment*, *705*, 135800. https://doi.org/10.1016/j.scitotenv.2019.135800

AlQahtani, F. S., AlShebly, M. M., Govindarajan, M., Senthilmurugan, S., Vijayan, P. & Benelli, G. (2017). Green and facile biosynthesis of silver nanocomposites using the aqueous extract of Rubus ellipticus leaves: toxicity and oviposition deterrent activity against Zika virus, malaria and filariasis mosquito vectors. *Journal of Asia-Pacific Entomology*, *20*(1), 157–164. https://doi.org/10.1016/j.aspen.2016.12.004

AlSalhi, M. S., Elumalai, K., Devanesan, S., Govindarajan, M., Krishnappa, K. & Maggi, F. (2020). The aromatic ginger Kaempferia galanga L.(Zingiberaceae) essential oil and its main compounds are effective larvicidal agents against Aedes vittatus and Anopheles maculatus without toxicity on the non-target aquatic fauna. *Industrial Crops and Products*, *158*, 113012. https://doi.org/10.1016/j.indcrop.2020.113012

Anjaneyulu, G., Nayak, V., Rao, U., Sateesh, T. & Mishra, K. (1999). Acute toxicity of neem oil to aquatic hemipteran predatory insect Notonecta sp. *Environment and Ecology*, *17*(1), 57–61.

Antwi, F. B. & Reddy, G. V. (2015). Toxicological effects of pyrethroids on non-target aquatic insects. *Environmental Toxicology and Pharmacology*, *40*(3), 915–923. https://doi.org/10.1016/j.etap.2015.09.023

Azarudeen, R. M. S. T., Govindarajan, M., Amsath, A., Kadaikunnan, S., Alharbi, N. S., Vijayan, P., Muthukumaran, U., et al. (2016). Size-controlled fabrication of silver nanoparticles using the Hedyotis puberula leaf extract: toxicity on mosquito vectors and impact on biological control agents. *RSC Advances*, *6*(99), 96573–96583. https://doi.org/10.1039/C6RA23208F

Azarudeen, R. M. S. T., Govindarajan, M., Amsath, A., Muthukumaran, U. & Benelli, G. (2017). Single-step biofabrication of silver nanocrystals using Naregamia alata: a cost effective and eco-friendly control tool in the fight against malaria, Zika virus and St. Louis encephalitis mosquito vectors. *Journal of Cluster Science*, *28*(1), 179–203. https://doi.org/10.1007/s10876-016-1067-y

Azarudeen, R. M. S. T., Govindarajan, M., AlShebly, M. M., AlQahtani, F. S., Amsath, A., Senthilmurugan, S., Vijayan, P., et al. (2017a). Size-controlled biofabrication of silver nanoparticles using the Merremia emarginata leaf extract: Toxicity on Anopheles stephensi, Aedes aegypti and Culex quinquefasciatus (Diptera: Culicidae) and non-target mosquito predators. *Journal of Asia-Pacific Entomology*, *20*(2), 359–366. https://doi.org/10.1016/j.aspen.2017.02.007

Azarudeen, R. M. S. T., Govindarajan, M., AlShebly, M. M., AlQahtani, F. S., Amsath, A. & Benelli, G. (2017b). One pot green synthesis of colloidal silver nanocrystals using the Ventilago maderaspatana leaf extract: acute toxicity on malaria, Zika virus and filariasis mosquito vectors. *Journal of Cluster Science*, *28*(1), 369–392. https://doi.org/10.1007/s10876-016-1103-y

Barahona, J., Millan, A. & Velasco, J. (2005). Population dynamics, growth and production of Sigara selecta (Fieber, 1848)(Hemiptera, Corixidae) in a Mediterranean hypersaline stream. *Freshwater Biology*, *50*(12), 2101–2113. https://doi.org/10.1111/j.1365-2427.2005.01463.x

Barmentlo, S. H., Schrama, M., van Bodegom, P. M., de Snoo, G. R., Musters, C. & Vijver, M. G. (2019). Neonicotinoids and fertilizers jointly structure naturally assembled freshwater macroinvertebrate communities. *Science of the Total Environment*, *691*, 36–44. https://doi.org/10.1016/j.scitotenv.2019.07.110

Benelli, G. (2016). Plant-mediated biosynthesis of nanoparticles as an emerging tool against mosquitoes of medical and veterinary importance: a review. *Parasitology Research*, *115*(1), 23–34. https://doi.org/10.1007/s00436-015-4800-9

Benelli, G. & Govindarajan, M. (2017). Green-synthesized mosquito oviposition attractants and ovicides: towards a nanoparticle-based “lure and kill” approach? *Journal of Cluster Science*, *28*(1), 287–308. https://doi.org/10.1007/s10876-016-1088-6

Benelli, G., Kadaikunnan, S., Alharbi, N. S. & Govindarajan, M. (2018a). Biophysical characterization of Acacia caesia-fabricated silver nanoparticles: effectiveness on mosquito vectors of public health relevance and impact on non-target aquatic biocontrol agents. *Environmental Science and Pollution Research*, *25*(11), 10228–10242. https://doi.org/10.1007/s11356-017-8482-y

Benelli, G., Govindarajan, M., Senthilmurugan, S., Vijayan, P., Kadaikunnan, S., Alharbi, N. S. & Khaled, J. M. (2018b). Fabrication of highly effective mosquito nanolarvicides using an Asian plant of ethno-pharmacological interest, Priyangu (Aglaia elaeagnoidea): toxicity on non-target mosquito natural enemies. *Environmental Science and Pollution Research*, *25*(11), 10283–10293. https://doi.org/10.1007/s11356-017-8898-4

Benelli, G., Rajeswary, M. & Govindarajan, M. (2018c). Towards green oviposition deterrents? Effectiveness of Syzygium lanceolatum (Myrtaceae) essential oil against six mosquito vectors and impact on four aquatic biological control agents. *Environmental Science and Pollution Research*, *25*(11), 10218–10227. https://doi.org/10.1007/s11356-016-8146-3

Benelli, G., Rajeswary, M., Vijayan, P., Senthilmurugan, S., Alharbi, N. S., Kadaikunnan, S., Khaled, J. M., et al. (2018d). Boswellia ovalifoliolata (Burseraceae) essential oil as an eco-friendly larvicide? Toxicity against six mosquito vectors of public health importance, non-target mosquito fishes, backswimmers, and water bugs. *Environmental Science and Pollution Research*, *25*(11), 10264–10271. https://doi.org/10.1007/s11356-017-8820-0

Bills, T. D., Marking, L. L. & Chandler Jr, J. H. (1977). Formalin: its toxicity to nontarget aquatic organisms, persistence, and counteraction. *US Department of the Interior, Fish and Wildlife Service,* 5–11.

Boda, P., Móra, A. & Csabai, Z. (2019). Aquatic macroinvertebrates from soda pans and adjacent wetland habitats of the Hungarian Puszta region with first records of four species from Hungary. *Spixiana, 42,* 263–282.

Booth, A., Moss, S. & Weyl, O. (2015). Effect of rotenone on gill-respiring and plastron-respiring insects. *African Journal of Aquatic Science*, *40*(1), 95–100. https://doi.org/10.2989/16085914.2014.986432

Brock, T. C. M., Roessink, I., Belgers, J. D. M., Bransen, F. & Maund, S. J. (2009). Impact of a benzoyl urea insecticide on aquatic macroinvertebrates in ditch mesocosms with and without non-sprayed sections. *Environmental Toxicology and Chemistry, 28*(10), 2191–2205. https://doi.org/10.1897/09-010.1

Carbonell, J. A., Gutiérrez-Cánovas, C., Bruno, D., Abellán, P., Velasco, J. & Millán, A. (2011). Ecological factors determining the distribution and assemblages of the aquatic Hemiptera (Gerromorpha & Nepomorpha) in the Segura River basin (Spain). *Limnetica, 30*(1), 59–70.

Carbonell, J., Millán, A. & Velasco, J. (2012). Concordance between realised and fundamental niches in three Iberian Sigara species (Hemiptera: Corixidae) along a gradient of salinity and anionic composition. *Freshwater Biology*, *57*(12), 2580–2590. https://doi.org/10.1111/fwb.12029

Case, T., TJ, C. & RK, W. (1978). Effects of the growth regulator methoprene on Culex tarsalis and non-target organisms in California rice fields. *Mosquito News*, *38*(2), 191–196.

Chandler Jr, J. H. & Marking, L. L. (1982). Toxicity of rotenone to selected aquatic invertebrates and frog larvae. *The Progressive Fish-Culturist*, *44*(2), 78–80. https://doi.org/10.1577/1548-8659(1982)44[78:TORTSA]2.0.CO;2

Crossland, N., Shires, S. & Bennett, D. (1982). Aquatic toxicology of cypermethrin. III. Fate and biological effects of spray drift deposits in fresh water adjacent to agricultural land. *Aquatic Toxicology*, *2*(5-6), 253–270. https://doi.org/10.1016/0166-445X(82)90015-7

Daam, M. A., Crum, S. J., Van den Brink, P. J. & Nogueira, A. J. (2008). Fate and effects of the insecticide chlorpyrifos in outdoor plankton-dominated microcosms in Thailand. *Environmental Toxicology and Chemistry: An International Journal*, *27*(12), 2530–2538. https://doi.org/10.1897/07-628.1

Dennett, J. A., Bernhardt, J. L. & Meisch, M. V. (2003). Operational note effects of fipronil and lambda-cyhalothrin against larval Anopheles quadrimaculatus and nontarget aquatic mosquito predators in Arkansas small rice plots. *Journal of the American Mosquito Control Association*, *19*(2), 172–174.

Esan, V., Mahboob, S., Al-Ghanim, K. A., Elanchezhiyan, C., Al-Misned, F., Ahmed, Z. & Govindarajan, M. (2020). Novel biogenic synthesis of silver nanoparticles using Alstonia venenata leaf extract: an enhanced mosquito larvicidal agent with negligible impact on important eco-biological fish and insects. *Journal of Cluster Science*, 1–9. https://doi.org/10.1007/s10876-020-01808-5

Esan, V., Mahboob, S., Al-Ghanim, K. A., Elanchezhiyan, C., Al-Misned, F., Ahmed, Z. & Govindarajan, M. (2021). Novel biogenic synthesis of silver nanoparticles using Alstonia venenata leaf extract: an enhanced mosquito larvicidal agent with negligible impact on important eco-biological fish and insects. *Journal of Cluster Science*, *32*(2), 489–497. https://doi.org/10.1007/s10876-020-01808-5

Fales, J., Spangler, P., Bodenstein, O., Mills, J., Durbin, J. & others. (1968). Laboratory and field evaluations of Abate against a backswimmer, Notonecta undulata Say (Hemiptera: Notonectidae). *Mosquito News*, *28*(1), 77–81.

Farlow, J., Breaud, T., Steelman, C. & Schilling, P. (1978). Effects of the Insect Growth Regulator Difluhenzuron on Non-Target Aquatic Populations in a Louisiana Intermediate Marsh. *Environmental Entomology*, *7*(2), 199–204. https://doi.org/10.1093/ee/7.2.199

Ferreira, F. A., Arcos, A. N., Maia, N. S., Sampaio, R., Costa, F. M., Rodrigues, I. B. & Tadei, W. P. (2020). Effects of diflubenzuron on associated insect fauna with Anopheles (Diptera: Culicidae) in laboratory, partial-field, and field conditions in the Central Amazon. *Anais da Academia Brasileira de Ciências*, *92*(1). e20180590 https://doi.org/10.1590/0001-3765202020180590

Fouzi, T. A., Youness, M., Guy, C., Ali, B. & Andrés, M. (2020). The alien boatman Trichocorixa verticalis verticalis (Hemiptera: Corixidae) is expanding in Morocco. *Limnetica*, *39*(1), 49–59.

Gallardo-Mayenco, A. (1994). Freshwater macroinvertebrate distribution in two basins with different salinity gradients (Guadalete and Guadaira river basins, south-western Spain). *International Journal of Salt Lake Research*, *3*(1), 75–91. https://doi.org/10.1007/BF01990644

Gerend, R. (2006). Micronecta scholtzi (Fieber, 1860) new to Luxembourg. With new records of three other rare aquatic heteropteran species (Insecta, Heteroptera). *Bulletin de la Société des Naturalistes Luxembourgeois, 106*, 63–65.

Golovatyuk, L. & Shitikov, V. (2016). Salinity tolerance of macrozoobenthic taxa in small rivers of the Lake Elton basin. *Russian Journal of Ecology*, *47*(6), 540–545. https://doi.org/10.1134/S1067413616060059

Govindarajan, M. & Benelli, G. (2016a). Eco-friendly larvicides from Indian plants: effectiveness of lavandulyl acetate and bicyclogermacrene on malaria, dengue and Japanese encephalitis mosquito vectors. *Ecotoxicology and Environmental Safety*, *133*, 395–402. https://doi.org/10.1016/j.ecoenv.2016.07.035

Govindarajan, M., Rajeswary, M., Muthukumaran, U., Hoti, S., Khater, H. F. & Benelli, G. (2016b). Single-step biosynthesis and characterization of silver nanoparticles using Zornia diphylla leaves: A potent eco-friendly tool against malaria and arbovirus vectors. *Journal of Photochemistry and Photobiology B: Biology*, *161*, 482–489. https://doi.org/10.1016/j.jphotobiol.2016.06.016

Govindarajan, M. & Benelli, G. (2016c). Facile biosynthesis of silver nanoparticles using Barleria cristata: mosquitocidal potential and biotoxicity on three non-target aquatic organisms. *Parasitology Research, 115*(3), 925–935. https://doi.org/10.1007/s00436-015-4817-0

Govindarajan, M., Rajeswary, M. & Benelli, G. (2016d). Chemical composition, toxicity and non-target effects of Pinus kesiya essential oil: an eco-friendly and novel larvicide against malaria, dengue and lymphatic filariasis mosquito vectors. *Ecotoxicology and Environmental Safety, 129*, 85–90. https://doi.org/10.1016/j.ecoenv.2016.03.007

Govindarajan, M., Kadaikunnan, S., Alharbi, N. S. & Benelli, G. (2016e). Acute toxicity and repellent activity of the Origanum scabrum Boiss. \& Heldr.(Lamiaceae) essential oil against four mosquito vectors of public health importance and its biosafety on non-target aquatic organisms. *Environmental Science and Pollution Research, 23*(22), 23228–23238. https://doi.org/10.1007/s11356-016-7568-2

Govindarajan, M., Rajeswary, M., Hoti, S., Murugan, K., Kovendan, K., Arivoli, S. & Benelli, G. (2016f). Clerodendrum chinense-mediated biofabrication of silver nanoparticles: Mosquitocidal potential and acute toxicity against non-target aquatic organisms. *Journal of Asia-Pacific Entomology, 19*(1), 51–58. https://doi.org/10.1016/j.aspen.2015.11.009

Govindarajan, M., Nicoletti, M. & Benelli, G. (2016g). Bio-physical characterization of poly-dispersed silver nanocrystals fabricated using Carissa spinarum: a potent tool against mosquito vectors. *Journal of Cluster Science, 27*(2), 745–761. https://doi.org/10.1007/s10876-016-0977-z

Govindarajan, M., Hoti, S. & Benelli, G. (2016h). Facile fabrication of eco-friendly nano-mosquitocides: biophysical characterization and effectiveness on neglected tropical mosquito vectors. *Enzyme and Microbial Technology, 95*, 155–163. https://doi.org/10.1016/j.enzmictec.2016.05.005

Govindarajan, M., Khater, H. F., Panneerselvam, C. & Benelli, G. (2016i). One-pot fabrication of silver nanocrystals using Nicandra physalodes: a novel route for mosquito vector control with moderate toxicity on non-target water bugs. *Research in Veterinary Science, 107*, 95–101. https://doi.org/10.1016/j.rvsc.2016.05.017

Govindarajan, M. & Benelli, G. (2017). A Facile One-Pot Synthesis of Eco-Friendly Nanoparticles Using Carissacarandas: Ovicidal and Larvicidal Potential on Malaria, Dengue and Filariasis Mosquito Vectors. *Journal of Cluster Science, 28*(1), 15–36. https://doi.org/10.1007/s10876-016-1035-6

Govindarajan, M., Kadaikunnan, S., Alharbi, N. S. & Benelli, G. (2017a). Single-step biological fabrication of colloidal silver nanoparticles using Hugonia mystax: larvicidal potential against Zika virus, dengue, and malaria vector mosquitoes. *Artificial Cells, Nanomedicine, and Biotechnology, 45*(7), 1317–1325. https://doi.org/10.1080/21691401.2016.1228664

Govindarajan, M., AlQahtani, F. S., AlShebly, M. M. & Benelli, G. (2017b). One-pot and eco-friendly synthesis of silver nanocrystals using Adiantum raddianum: toxicity against mosquito vectors of medical and veterinary importance. *Journal of Applied Biomedicine, 15*(2), 87–95. https://doi.org/10.1016/j.jab.2016.10.004

Govindarajan, M., Rajeswary, M., Senthilmurugan, S., Vijayan, P., Alharbi, N. S., Kadaikunnan, S., Khaled, J. M., et al. (2018a). Larvicidal activity of the essential oil from Amomum subulatum Roxb.(Zingiberaceae) against Anopheles subpictus, Aedes albopictus and Culex tritaeniorhynchus (Diptera: Culicidae), and non-target impact on four mosquito natural enemies. *Physiological and Molecular Plant Pathology*, *101*, 219–224. https://doi.org/10.1016/j.pmpp.2017.01.003

Govindarajan, M., Rajeswary, M., Senthilmurugan, S., Vijayan, P., Alharbi, N. S., Kadaikunnan, S., Khaled, J. M., et al. (2018b). Curzerene, trans-beta-elemenone, and gamma-elemene as effective larvicides against Anopheles subpictus, Aedes albopictus, and Culex tritaeniorhynchus: toxicity on non-target aquatic predators. *Environmental Science and Pollution Research*, *25*(11), 10272–10282. https://doi.org/10.1007/s11356-017-8822-y

Grigarick, A., Webster, R., Meyer, R., Zalom, F., Smith, K. & others. (1990). Effect of pesticide treatments on nontarget organisms in California rice paddies. I. Impact of triphenyltin hydroxide. II. Impact of diflubenzuron and triflumuron. *Hilgardia*, *58*(1), 1–40. https://doi.org/10.3733/hilg.v58n01p036

Gunter, G. & Christmas, J. (1959). Corixid insects as part of the offshore fauna of the sea. *Ecology*, *40*(4), 724–725. https://doi.org/10.2307/1929829

Hall, B., Rosenberg, D. & Wiens, A. (1998). Methyl mercury in aquatic insects from an experimental reservoir. *Canadian Journal of Fisheries and Aquatic Sciences*, *55*(9), 2036–2047. https://doi.org/10.1139/f98-079

Hashimoto, K., Kasai, A., Hayasaka, D., Goka, K. & Hayashi, T. I. (2020). Long-term monitoring reveals among-year consistency in the ecological impacts of insecticides on animal communities in paddies. *Ecological Indicators*, *113*, 106227. https://doi.org/10.1016/j.ecolind.2020.106227

Hayasaka, D., Kobashi, K. & Hashimoto, K. (2019). Community responses of aquatic insects in paddy mesocosms to repeated exposures of the neonicotinoids imidacloprid and dinotefuran. *Ecotoxicology and Environmental Safety*, *175*, 272–281. https://doi.org/10.1016/j.ecoenv.2019.03.051

Hoi, T. M., Huong, L. T., Chinh, H. V., Hau, D. V., Satyal, P., Tai, T. A., Dai, D. N., et al. (2020). Essential Oil Compositions of Three Invasive Conyza Species Collected in Vietnam and Their Larvicidal Activities against Aedes aegypti, Aedes albopictus, and Culex quinquefasciatus. *Molecules*, *25*(19), 4576. https://doi.org/10.3390/molecules25194576

Hose, G. C., Lim, R. P., Hyne, R. V. & Pablo, F. (2002). A pulse of endosulfan-contaminated sediment affects macroinvertebrates in artificial streams. *Ecotoxicology and Environmental Safety*, *51*(1), 44–52. https://doi.org/10.1006/eesa.2001.2127

Kanaoka, A., Kodama, H., Yamaguchi, R., Konno, T., Kajihara, O. & Maekawa, S. (1994). Influence of buprofezin on natural enemies and non-target insects in the paddy field. *Journal of Pesticide Science*, *19*(4), 309–312. https://doi.org/10.1584/jpestics.19.4_309

Kelts, L. J. (1979). Ecology of a tidal marsh corixid, Trichocorixa verticalis (Insecta, Hemiptera). *Hydrobiologia*, *64*(1), 37–57. https://doi.org/10.1007/BF00015451

Knowles, J. N. & Williams, W. (1973). Salinity range and osmoregulatory ability of corixids (Hemiptera: Heteroptera) in south-east Australian inland waters. *Marine and Freshwater Research*, *24*(3), 297–302. https://doi.org/10.1071/MF9730297

Konar, S. (1970). Toxicity of heptachlor to aquatic life. *Journal (Water Pollution Control Federation), 42*(8), R299–R303.

Koodalingam, A., Mullainadhan, P. & Arumugam, M. (2009). Antimosquito activity of aqueous kernel extract of soapnut Sapindus emarginatus: impact on various developmental stages of three vector mosquito species and nontarget aquatic insects. *Parasitology Research, 105*(5), 1425. https://doi.org/10.1007/s00436-009-1574-y

Lahr, J., Diallo, A. O., Gadji, B., Diouf, P. S., Bedaux, J. J., Badji, A., Ndour, K. B., et al. (2000). Ecological effects of experimental insecticide applications on invertebrates in Sahelian temporary ponds. *Environmental Toxicology and Chemistry: An International Journal*, *19*(5), 1278–1289. https://doi.org/10.1002/etc.5620190509

Lawler, S., Dritz, D. & Jensen, T. (2000). Effects of sustained-release methoprene and a combined formulation of liquid methoprene and Bacillus thuringiensis israelensis on insects in salt marshes. *Archives of Environmental Contamination and Toxicology* , *39*(2), 177–182. https://doi.org/10.1007/s002440010094

Marking, L. L. (1974). Toxicity of 2-(digeranylamino)-ethanol, a candidate selective fish toxicant. *Transactions of the American Fisheries Society*, *103*(4), 736–742. https://doi.org/10.1577/1548-8659(1974)103%3C736:TODACS%3E2.0.CO;2

Maund, S. J., Hamer, M. J., Warinton, J. S. & Kedwards, T. J. (1998). Aquatic ecotoxicology of the pyrethroid insecticide lambda-cyhalothrin: Considerations for higher-tier aquatic risk assessment. *Pesticide Science*, *54*(4), 408–417. https://doi.org/10.1002/(SICI)1096-9063(199812)54:4%3C408::AID-PS843%3E3.0.CO;2-T

Maund, S., Peither, A., Taylor, E., Jüttner, I., Beyerle-Pfnür, R., Lay, J. & Pascoe, D. (1992). Toxicity of lindane to freshwater insect larvae in compartments of an experimental pond. *Ecotoxicology and Environmental Safety*, *23*(1), 76–88. https://doi.org/10.1016/0147-6513(92)90023-V

Melaas, C. L., Zimmer, K. D., Butler, M. G. & Hanson, M. A. (2001). Effects of rotenone on aquatic invertebrate communities in prairie wetlands. *Hydrobiologia*, *459*(1-3), 177–186. https://doi.org/10.1023/A:1012514124430

Mills, J., Fales, J., Durbin, J. & others. (1969). Comparison of the effect of six pyrethroids against a backswimmer, Notonecta undulata Say. *Mosquito News*, *29*(4), 690–691.

Miura, T. & Takahashi, R. (1973). Insect developmental inhibitors. 3. Effects on nontarget aquatic organisms. *Journal of Economic Entomology*, *66*(4), 917–922. https://doi.org/10.1093/jee/66.4.917

Murata, K. & Tanaka, K. (2020). Insecticide susceptibilities of Hydrometra species (Hemiptera: Hydrometridae), including an endangered species, inhabiting paddy fields in Japan. *Applied Entomology and Zoology*, *55*(4), 395–403. https://doi.org/10.1007/s13355-020-00695-6

Naqvi, S. M. & De la Cruz, A. A. (1973). Mirex incorporation in the environment: toxicity in selected freshwater organisms. *Bulletin of Environmental Contamination and Toxicology* , *10*(5), 305–308. https://doi.org/10.1007/BF01684821

Nummelin, M., Lodenius, M. & Tulisalo, E. (1997). Water striders (Heteroptera, Gerridae) as bioindicators of heavy metal pollution. *Entomologica Fennica*, *8*(4), 185–191. https://doi.org/10.33338/ef.83942

Rajeswary, M., Govindarajan, M., Alharbi, N. S., Kadaikunnan, S., Khaled, J. M. & Benelli, G. (2018). Zingiber cernuum (Zingiberaceae) essential oil as effective larvicide and oviposition deterrent on six mosquito vectors, with little non-target toxicity on four aquatic mosquito predators. *Environmental Science and Pollution Research*, *25*(11), 10307–10316. https://doi.org/10.1007/s11356-017-9093-3

Rawani, A. (2020). Larvicidal efficacy of the mature leaf extract of Cajanus cajan against the vector of Japanese encephalitis. *International Journal of Tropical Insect Science*, 1–7. https://doi.org/10.1007/s42690-020-00300-x

Rawani, A., Ghosh, A. & Chandra, G. (2013). Mosquito larvicidal and antimicrobial activity of synthesized nano-crystalline silver particles using leaves and green berry extract of Solanum nigrum L.(Solanaceae: Solanales). *Acta Tropica*, *128*(3), 613–622. https://doi.org/10.1016/j.actatropica.2013.09.007

Roberts, L., Roberts, D., Miller, T., Nelson, L., Young, W. & others. (1973). Polymer formulations of mosquito larvicides. III. Effects of a polyethylene formulation of chlorpyrifos on non-target populations naturally infesting artificial field pools. *Mosquito News*, *33*(2), 165–172.

Roessink, I., Merga, L. B., Zweers, H. J. & Van den Brink, P. J. (2013). The neonicotinoid imidacloprid shows high chronic toxicity to mayfly nymphs. *Environmental Toxicology and Chemistry*, *32*(5), 1096–1100. https://doi.org/10.1002/etc.2201

Rubach, M. N., Crum, S. J. H. & Van den Brink, P. J. (2011). Variability in the dynamics of mortality and immobility responses of freshwater arthropods exposed to chlorpyrifos. *Archives of Environmental Contamination and Toxicology* , *60*(4), 708–721. https://doi.org/10.1007/s00244-010-9582-6

Saha, S. & Kaviraj, A. (2008). Acute toxicity of synthetic pyrethroid cypermethrin to some freshwater organisms. *Bulletin of Environmental Contamination and Toxicology* , *80*(1), 49–52. https://doi.org/10.1007/s00128-007-9314-4

Samidurai, K. & Mathew, N. (2014). Bioassay guided fractionation and GC-MS analysis of Euphorbia lactea extract for mosquito larvicidal activity. *International Journal of Pharmacy and Pharmaceutical Sciences*, *6*, 344–347.

Samsøe-Petersen, L., Gustavson, K., Madsen, T., Mogensen, B. B., Lassen, P., Skjernov, K., Christoffersen, K., et al. (2001). Fate and effects of esfenvalerate in agricultural ponds. *Environmental Toxicology and Chemistry*, *20*(7), 1570–1578. https://doi.org/10.1002/etc.5620200722

Scheuhammer, A., McNicol, D., Mallory, M. & Kerekes, J. (1997). Relationships between lake chemistry and calcium and trace metal concentrations of aquatic invertebrates eaten by breeding insectivorous waterfowl. *Environmental Pollution*, *96*(2), 235–247. https://doi.org/10.1016/S0269-7491(97)00032-8

Ser, Ö. & Cetin, H. (2015). Toxicity of mosquito larvicides on non-target mosquito predator insect, backswimmer (Notonecta sp.). *Fresenius Environmental Bulletin*, *24*(1), 311–316.

Shaw, J. L. & Manning, J. P. (1996). Evaluating macroinvertebrate population and community level effects in outdoor microcosms: use of in situ bioassays and multivariate analysis. *Environmental Toxicology and Chemistry: An International Journal*, *15*(5), 608–617. https://doi.org/10.1002/etc.5620150502

Sherratt, T. N., Roberts, G., Williams, P., Whitfield, M., Biggs, J., Shillabeer, N. & Maund, S. J. (1999). A life-history approach to predicting the recovery of aquatic invertebrate populations after exposure to xenobiotic chemicals. *Environmental Toxicology and Chemistry: An International Journal*, *18*(11), 2512–2518. https://doi.org/10.1002/etc.5620181118

Shires, S. & Bennett, D. (1985). Contamination and effects in freshwater ditches resulting from an aerial application of cypermethrin. *Ecotoxicology and Environmental Safety, 9*(2), 145–158. https://doi.org/10.1016/0147-6513(85)90017-X

Shoba, V., Elanchezhiyan, C., Hemalatha, S. & Selvisabanayakam, S. (2011). Sublethal effect of phytopesticide nimbecidine on biochemical changes in the adult male insect Sphaerodema rusticum (Heteroptera: Belostomatidae). *International Journal* of *Research in Pharmaceutical Sciences*, *2*(1), 12–17.

Siling, R. & Urbani, G. (2016). Do lake littoral benthic invertebrates respond differently to eutrophication, hydromorphological alteration, land use and fish stocking? *Knowledge & Management of Aquatic Ecosystems*, (417), 35. https://doi.org/10.1051/kmae/2016022

Singha, S., Adhikari, U., Ghosh, A. & Chandra, G. (2012). Mosquito larvicidal potentiality of Holoptelea integrifolia leaf extract against Japanese encephalitis vector, Culex vishnui group. *Journal of Mosquito Research*, *2*(4), 25–31. https://doi.org/10.5376/jmr.2012.02.0004

Sivagnaname, N. & Kalyanasundaram, M. (2004). Laboratory evaluation of methanolic extract of Atlantia monophylla (Family: Rutaceae) against immature stages of mosquitoes and non-target organisms. *Memórias do Instituto Oswaldo Cruz*, *99*(1), 115–118. https://doi.org/10.1590/S0074-02762004000100021

Steelman, C., Farlow, J., Breaud, T. & Schilling, P. (1975). Effects of the growth regulators on Psorophora columbiae(Dyar and Knab) and non-target aquatic insect species in rice fields. *Mosquito News*, *35*(1), 67–76.

Steelman, C. & Schilling, P. (1972). Effects of a juvenile hormone mimic on Psorophora confinnis (Lynch-Arribalzaga) and non-target aquatic insects. *Mosquito News*, *32*(3) 350–354.

Stephenson, R. (1982). Aquatic toxicology of cypermethrin. I. Acute toxicity to some freshwater fish and invertebrates in laboratory tests. *Aquatic Toxicology*, *2*(3), 175–185. https://doi.org/10.1016/0166-445X(82)90014-5

Sundaram, K. M., Holmes, S. B., Kreutzweiser, D. P., Sundaram, A. & Kingsbury, P. D. (1991). Environmental persistence and impact of diflubenzuron in a forest aquatic environment following aerial application. *Archives of Environmental Contamination and Toxicology*, *20*(3), 313–324. https://doi.org/10.1007/BF01064396

Takahashi, R., Wilder, W. & Miura, T. (1984). Field evaluations of ISA-20 E for mosquito control and effects on aquatic nontarget arthropods in experimental plots. *Mosquito News*, *44*(3), 363–367.

Thanigaivel, A., Vasantha-Srinivasan, P., Senthil-Nathan, S., Edwin, E.-S., Ponsankar, A., Chellappandian, M., Selin-Rani, S., et al. (2017). Impact of Terminalia chebula Retz. against Aedes aegypti L. and non-target aquatic predatory insects. *Ecotoxicology and Environmental Safety*, *137*, 210–217. https://doi.org/10.1016/j.ecoenv.2016.11.004

Tsui, M. T. K., Finlay, J. C. & Nater, E. A. (2009). Mercury bioaccumulation in a stream network. *Environmental Science & Technology*, *43*(18), 7016–7022. https://doi.org/10.1021/es901525w

Tsui, M. T.-K., Blum, J. D., Finlay, J. C., Balogh, S. J., Nollet, Y. H., Palen, W. J. & Power, M. E. (2014). Variation in terrestrial and aquatic sources of methylmercury in stream predators as revealed by stable mercury isotopes. *Environmental Science & Technology*, *48*(17), 10128–10135. https://doi.org/10.1021/es500517s

Van Breukelen, S. & Brock, T. (1993). Response of a macro-invertebrate community to insecticide application in replicated freshwater microcosms with emphasis on the use of principal component analysis. *Science of the Total Environment*, *134*, 1047–1058. https://doi.org/10.1016/S0048-9697(05)80110-X

Van den Brink, P. J., Van Smeden, J. M., Bekele, R. S., Dierick, W., De Gelder, D. M., Noteboom, M. & Roessink, I. (2016). Acute and chronic toxicity of neonicotinoids to nymphs of a mayfly species and some notes on seasonal differences. *Environmental Toxicology and Chemistry*, *35*(1), 128–133. https://doi.org/10.1002/etc.3152

Van Wijngaarden, R. P. A., Barber, I. & Brock, T. C. M. (2009). Effects of the pyrethroid insecticide gamma-cyhalothrin on aquatic invertebrates in laboratory and outdoor microcosm tests. *Ecotoxicology*, *18*(2), 211–224. https://doi.org/10.1007/s10646-008-0274-1

Verberk, W. C. E. P., Van Kleef, H. H., Dijkman, M., Van Hoek, P., Spierenburg, P. & Esselink, H. (2005). Seasonal changes on two different spatial scales: response of aquatic invertebrates to water body and microhabitat. *Insect Science*, *12*(4), 263–280. https://doi.org/10.1111/j.1005-295X.2005.00033.x

Zinchenko, T. & Golovatyuk, L. (2013). Salinity tolerance of macroinvertebrates in stream waters. *Arid Ecosystems*, *3*(3), 113–121. https://doi.org/10.1134/S2079096113030116
